# The global, regional, and national burden of secondhand smoke-related tracheal, bronchus, and lung cancer: Results from the Global Burden of Disease study 2021

**DOI:** 10.18332/tid/205049

**Published:** 2025-06-12

**Authors:** Jinfeng Yao, Liming Xia, Huiqin Lu, Tao Zhang, Tongfei Zhang, Guo Chen

**Affiliations:** 1 Department of Oncology, Shuguang Anhui Hospital Affiliated to Shanghai University of Traditional Chinese Medicine (The First Affiliated Hospital West District of Anhui University of Chinese Medicine), Hefei, China; 2 Department of Oncology, The First Affiliated Hospital of Anhui University of Chinese Medicine, Hefei, China

**Keywords:** TBL cancer, secondhand smoke, GBD 2021

## Abstract

**INTRODUCTION:**

This study analyzes the global, regional, and national health burden of secondhand smoke-related tracheal, bronchus, and lung (TBL) cancer from 1990 to 2021.

**METHODS:**

This is a secondary dataset analysis of the GBD dataset. First, the number and age-standardized rate (ASR) per 100000 population of deaths and disability-adjusted life years (DALY) related to TBL cancer due to secondhand smoke in 2021 were analyzed at multiple levels. The temporal trends in disease burden between 1990 and 2021 were then analyzed by a linear regression model.

**RESULTS:**

Globally, the number of deaths and DALYs from secondhand smoke-related TBL cancer increased from 57.6 thousand and 1598.9 thousand in 1990 to 97.9 thousand and 2355.9 thousand in 2021. Men faced higher risks, with 56.8 thousand deaths and 1359.6 thousand DALYs in 2021. The highest number of deaths and DALYs occurred in the age groups of 70–74 years (15731; 95% UI: 1787–30495) and 65–69 years (380606; 95% UI: 47383–717297), respectively. Disease burden varies widely across sociodemographic index (SDI) regions, GBD regions, and countries. In 2021, the high-middle SDI region had the highest ASRs of deaths (1.96; 95% UI: 0.23–3.67) and DALYs (47.2; 95% UI: 5.65–87.54), and the highest number of deaths (39124; 95% UI: 4613–73341) and DALYs (936577; 95% UI: 111577–1736627). ASR for deaths and DALYs was highest in East Asia (2.75; 95% UI: 0.34–5.18 and 62.42; 95% UI: 7.79–116.4). Among the countries, China has the highest number of deaths (58034; 95% UI: 7170–109625) and DALYs (1359730; 95% UI: 170188–2537368), and Montenegro has the highest ASR of deaths (3.45; 95% UI: 0.38–7.06) and DALYs (84.34; 95% UI: 9.36–170.96).

**CONCLUSIONS:**

This study describes the disease burden of secondhand smoke-related TBL cancer, emphasizing secondhand smoke is a non-negligible risk factor for TBL cancer. The findings of this study can serve as a basis for formulating targeted tobacco control policies, which could significantly contribute to reducing the global burden of TBL cancer.

## INTRODUCTION

Tracheal, bronchus, and lung (TBL) cancer represent a major global public health challenge. The Cancer Tomorrow online tool of the GLOBOCAN indicates that in 2022, lung cancer accounted for the highest incidence (2480301 new cases; 12.4% of total cases) and mortality (1817172 deaths; 18.7% of total deaths) among all cancers worldwide, and the incident cases are estimated to increase by 77% by 2050^1^. Among all malignancies, TBL cancer has the highest economic burden, with estimated costs reaching US$3.9 trillion in 2017. Projections indicate it will continue to account for the largest proportion (15.4%) of global cancer-related economic costs from 2020 to 2050, representing the most significant cancer-related burden in most countries, including China and the United States^2^.The clinical outcomes of early-stage TBL cancer patients is significantly better than that of middle-stage and late-stage patients^3^. Therefore, early detection, screening and treatment can effectively lower the mortality rate of TBL cancer and reduce the burden of TBL cancer.

Risk factors for TBL cancer include three major categories: environmental factors, lifestyle, and occupational exposure. Environmental risk factors comprise ambient particulate matter pollution, household air pollution from solid fuel, and residential radon. Behavioral determinants positively associated with TBL cancer burden include diet low in fruits, high fasting plasma glucose, smoking and secondhand smoke. Occupational exposure to diesel engine exhaust, asbestos and radioactive substances is associated with the development of TBL cancer. These factors act on the human body through different mechanisms, increasing the incidence of TBL cancer and adding to the burden of TBL cancer^4,5^.

Both active smoking and secondhand smoke are important risk factors for TBL cancer, and long-term exposure to secondhand smoke significantly increases the risk of TBL cancer among never smokers^4^. Studies have shown that secondhand smoke contains a variety of carcinogens, such as benzopyrene and nitrosamine, which can damage the DNA of lung cells, leading to gene mutations, thus inducing lung cancer^6^. Authoritative reports show that secondhand smoke exposure causes a variety of cancers and even premature death^7,8^.

Previous studies have extensively reported on the burden of TBL cancer attributed to smoking^9,10^. However, there is a relative scarcity of research on the disease burden attributed to secondhand smoke. Consequently, it is imperative to assess the comprehensive burden of TBL cancer caused by secondhand smoke and to update the epidemiological data with the most recent information.

This study assesses the global, regional, and national burden of secondhand smoke-related TBL cancer by analyzing mortality, and disability-adjusted life years (DALYs) from 1990 to 2021. With the aim of offering valuable perspectives on the development of secondhand smoke-related TBL cancer epidemiology, it will inform TBL cancer prevention strategies to mitigate the escalating burden of this disease.

## METHODS

### Data sources

This is a secondary dataset analysis of the GBD dataset. All data were downloaded from the Global Health Data Exchange (GHDx) query tool (https://ghdx.healthdata.org/gbd-2021)^11^. The Global Burden of Disease (GBD) 2021 study collects data from household surveys, censuses, vital statistics, disease registries, health service use, disease notifications, satellite imaging, and other sources for modeling and estimating the burden of each country and region in several scenarios from 1990 to 2021 using Bayesian meta regression modeling tools^2^. GBD provides comprehensive estimates of deaths and disability-adjusted life years by cause, age, sex, and location^13^. The GBD study used a comparative risk assessment methodology to estimate the attributable burden of secondhand smoke-related tracheal, bronchus, and lung cancer using data from multiple sources^14,15^.

### Definitions

TBL cancer is defined as tumors within the trachea, bronchus, or lung, as classified in the International Classification of Diseases (ICD) 10 by codes C33 and C34-C34^16^. The ICD-10 is the current diagnostic standard and the most comprehensive classification.

The sociodemographic index (SDI) functions as a composite indicator that evaluates regional development level such as education level, fertility rate, and per capita income^17^. This standardized metric, ranges from 0 to 1, uses higher values to indicate greater socio-economic development. As reported in GBD 2021 study, 204 countries and territories were stratified into five SDI groups: low (≤0.454743), low-middle (0.454743–0.607679), middle (0.607679–0.689504), high-middle (0.689504–0.805129), and high SDI (>0.805129)^17^.

Disability-adjusted life years (DALYs) quantify the overall disease burden by combining years of life lost (YLLs) and years lived with disability (YLDs), and represent the total years of healthy life lost from the onset of a disease to death, expressed^16^ as DALYs = YLLs + YLDs.

Age-standardized rates (ASRs) were used to compare incidence, prevalence, mortality, and DALYs across countries or regions with varying age structures and demographic characteristics.

### Statistical analysis

First, we report the number of secondhand smoke-related tracheal, bronchus, and lung cancer deaths and DALYs, and corresponding age-standardized rates (ASR) per 100000 population for 1990 and 2021. The analysis was conducted globally and stratified by subtypes, including GBD region, country, sociodemographic index (SDI) region (constructed based on three indicators ranging from 0 to 1, with larger SDIs indicating more developed^18^), gender, and age group. Then, we analyzed global and different subtype trends from 1990 to 2021 to study the dynamics of the disease burden. Finally, we performed a hierarchical cluster analysis by calculating the estimated annual percentage change (EAPC) values, dividing the 54 GBD regions into four groups, including minor increase, remained stable or minor decrease, significant increase and significant decrease.

In this study, all count and rates are presented with 95% uncertainty intervals (UIs)^12^ (generated using the 2.5th and 97.5th percentile ordered 1000 draws of the posterior distribution). All the rates are reported per 100000 population. EAPC values are given with 95% confidence intervals (CIs). All statistical analyses and visualizations were performed using the R statistical software program (V.4.0.2) and p<0.05 was considered a statistically significant difference.

## RESULTS

### The disease burden of secondhand smokerelated tracheal, bronchus, and lung cancer in 2021

An estimated 97911 (95% UI: 11955–184913 million) deaths from secondhand smoke-related tracheal, bronchus, and lung cancer worldwide in 2021, with an age-standardized death rate (ASDR) of 1.14 deaths per 100000 (95% UI: 0.14–2.15) (Table 1). In addition, the number of DALYs was 2355866 (95% UI: 290211–4442996), with an age-standardized DALYs rate (ASDAR) of 26.93 per 100000 (95% UI: 3.32–50.83) (Table 2).

**Table 1:** Deaths and age-standardized deaths rate of secondhand smoke-related TBL cancer, globally, by sex and age, and SDI region, in 1990 and 2021, and trends from 1990 to 2021

	*Deaths (95% UI)* *1990*	*Age-standardized* *deaths rate/100000* *(95% UI)* *1990*	*Deaths (95% UI)* *2021*	*Age-standardized* *deaths rate/100000* *(95% UI)* *2021*	*EAPC (95% CI)*
**Global**	57618 (7083–107842)	1.45 (0.18–2.72)	97911 (11955–184913)	1.14 (0.14–2.15)	-0.88 (-0.94 – -0.82)
**Sex**					
Female	20854 (2762–37840)	0.98 (0.13–1.78)	41063 (5536–78059)	0.89 (0.12–1.69)	-0.57 (-0.65 – -0.49)
Male	36764 (4318–69861)	2.03 (0.24–3.86)	56848 (6655–109071)	1.44 (0.17–2.78)	-1.07 (-1.15 – -1)
**Age** (years)					
25–29	204 (27–379)	0.05 (0.01–0.09)	159 (21–292)	0.03 (0–0.05)	-1.82 (-2.04 – -1.6)
30–34	390 (52–711)	0.1 (0.01–0.18)	392 (53–767)	0.06 (0.01–0.13)	-1.91 (-2.15 – -1.67)
35–39	872 (109–1606)	0.25 (0.03–0.46)	713 (90–1356)	0.13 (0.02–0.24)	-2.57 (-2.76 – -2.38)
40–44	1673 (213–3084)	0.58 (0.07–1.08)	1443 (187–2734)	0.29 (0.04–0.55)	-2.5 (-2.68 – -2.33)
45–49	2852 (357–5334)	1.23 (0.15–2.3)	3091 (411–5894)	0.65 (0.09–1.24)	-2.14 (-2.33 – -1.94)
50–54	5510 (661–10198)	2.59 (0.31–4.8)	6573 (818–12153)	1.48 (0.18–2.73)	-1.96 (-2.12 – -1.8)
55–59	8212 (1015–15334)	4.43 (0.55–8.28)	10490 (1374–19668)	2.65 (0.35–4.97)	-1.73 (-1.83 – -1.63)
60–64	9873 (1234–18691)	6.15 (0.77–11.64)	12288 (1593–23248)	3.84 (0.5–7.26)	-1.36 (-1.45 – -1.26)
65–69	9238 (1121–17234)	7.47 (0.91–13.94)	15495 (1929–29214)	5.62 (0.7–10.59)	-1.16 (-1.25 – -1.07)
70–74	7508 (927–14291)	8.87 (1.1–16.88)	15731 (1787–30495)	7.64 (0.87–14.81)	-0.71 (-0.82 – -0.6)
75–79	5959 (738–11246)	9.68 (1.2–18.27)	12741 (1624–25098)	9.66 (1.23–19.03)	-0.1 (-0.23–0.03)
80–84	3398 (403–6501)	9.61 (1.14–18.38)	9830 (1225–19375)	11.22 (1.4–22.12)	0.56 (0.42–0.7)
85–89	1469 (181–2824)	9.72 (1.2–18.69)	6203 (747–12124)	13.57 (1.63–26.52)	0.99 (0.86–1.13)
90–94	387 (46–743)	9.03 (1.07–17.34)	2197 (288–4377)	12.28 (1.61–24.47)	0.94 (0.88–0.99)
≥95	72 (9–140)	7.08 (0.84–13.75)	564 (73–1155)	10.34 (1.34–21.19)	1.02 (0.87–1.17)
**SDI region**					
High-middle	21702 (2611–40729)	2.16 (0.26–4.04)	39124 (4613–73341)	1.96 (0.23–3.67)	-0.36 (-0.46 – -0.25)
High	18917 (2323–36155)	1.74 (0.21–3.33)	16945 (2216–32743)	0.82 (0.11–1.58)	-2.57 (-2.67 – -2.46)
Low-middle	2350 (311–4440)	0.39 (0.05–0.74)	5478 (699–10459)	0.38 (0.05–0.73)	-0.13 (-0.17 – -0.09)
Low	374 (43–740)	0.17 (0.02–0.33)	770 (94–1556)	0.15 (0.02–0.31)	-0.43 (-0.52 – -0.35)
Middle	14192 (1799-25709)	1.41 (0.18–2.56)	35511 (4497–67457)	1.36 (0.17–2.58)	-0.24 (-0.3 – -0.18)

TBL: tracheal, bronchus, and lung.

**Table 2:** DALYs and age-standardized DALYs rate (ASDAR)of secondhand smoke-related TBL cancer, globally, by sex and age, and SDI region, in 1990 and 2021, and ASDAR trends from 1990 to 2021

	*DALYs (95% UI)* *1990*	*Age-standardized* *DALYs rate/100000* *(95% UI)* *1990*	*DALYs (95% UI)* *2021*	*Age-standardized* *DALYs rate/100000* *(95% UI)* *2021*	*EAPC (95% CI)*
**Global**	1598871 (196922–2982788)	38.4 (4.72–71.68)	2355866 (290211–4442996)	26.93 (3.32–50.83)	-1.25 (-1.31 – -1.19)
**Sex**					
Female	577953 (76274–1045073)	26.56 (3.5–48.03)	996309 (135988–1907079)	21.81 (2.98–41.75)	–0.89 (-0.97 – -0.81)
Male	1020918 (120602–1938069)	51.88 (6.11–98.51)	1359557 (158640–2596453)	32.88 (3.85–62.82)	-1.48 (-1.55 – -1.4)
**Age** (years)					
25–29	12872 (1696–23888)	2.91 (0.38–5.4)	10048 (1309–18372)	1.71 (0.22–3.12)	-1.82 (-2.04 – -1.6)
30–34	22611 (3001–41191)	5.87 (0.78–10.69)	22752 (3059–44516)	3.76 (0.51–7.36)	-1.91 (-2.15 – -1.67)
35–39	46342 (5794–85284)	13.16 (1.64–24.21)	37932 (4761–72164)	6.76 (0.85–12.87)	-2.57 (-2.76 – -2.38)
40–44	80650 (10272–148680)	28.15 (3.59–51.9)	69534 (9009–131730)	13.9 (1.8–26.33)	-2.51 (-2.69 – -2.33)
45–49	123239 (15411–230486)	53.08 (6.64–99.26)	133648 (17758–254888)	28.23 (3.75–53.83)	-2.14 (-2.33 – -1.94)
50–54	211821 (25408–391862)	99.65 (11.95–184.34)	253050 (31482–467824)	56.87 (7.08–105.15)	-1.96 (-2.12 – -1.79)
55–59	277096 (34227–517440)	149.62 (18.48–279.4)	355140 (46469–666205)	89.74 (11.74–168.35)	-1.72 (-1.83 – -1.62)
60–64	287476 (35899–544069)	178.99 (22.35–338.75)	358037 (46381–678102)	111.87 (14.49–211.88)	-1.35 (-1.44 – -1.25)
65–69	226793 (27500–423309)	183.48 (22.25–342.46)	380606 (47383–717297)	137.98 (17.18–260.04)	-1.15 (-1.24 – -1.06)
70–74	151674 (18709–288562)	179.15 (22.1–340.84)	318403 (36124–616739)	154.69 (17.55-299.62)	-0.71 (-0.82 – -0.6)
75–79	96335 (11906–181899)	156.5 (19.34–295.5)	206073 (26270–405449)	156.25 (19.92–307.43)	-0.11 (-0.24–0.03)
80–84	43132 (5108–82579)	121.93 (14.44–233.43)	124319 (15511–245162)	141.94 (17.71–279.92)	0.55 (0.41–0.69)
85–89	14844 (1828–28609)	98.24 (12.1–189.32)	62440 (7523–122160)	136.57 (16.45–267.18)	0.98 (0.85–1.12)
90–94	3393 (401–6532)	79.18 (9.36–152.43)	19286 (2524–38423)	107.81 (14.11–214.78)	0.94 (0.89–0.99)
≥95	596 (70–1156)	58.5 (6.92–113.59)	4600 (593–9429)	84.39 (10.89–172.99)	0.98 (0.82–1.13)
**SDI region**					
High-middle	609304 (73612–1146504)	58.69 (7.08–110.37)	936577 (111577–1736627)	47.2 (5.65–87.54)	-0.78 (-0.89 – -0.67)
High	507723 (62348–966857)	48.12 (5.91–91.54)	380285 (49959–738240)	19.99 (2.63–38.76)	-2.93 (-3.04 – -2.81)
Low-middle	67474 (8771–127412)	10.15 (1.33–19.21)	152292 (19444–291023)	9.9 (1.26–18.89)	-0.13 (-0.17 – -0.09)
Low	10837 (1223–21437)	4.37 (0.5–8.65)	22327 (2712–45068)	3.96 (0.48–8.01)	-0.49 (-0.58 – -0.4)
Middle	401195 (50957–727467)	35.8 (4.54–64.87)	862344 (110218–1626035)	31.13 (3.97–58.93)	-0.58 (-0.63 – -0.52)

DALYs: disability-adjusted life years.

From the perspective of gender, the burden of men exceeded that of women, both in terms of the number of deaths and the number of DALYs (Supplementary file: Figure 1, and Tables 1 and 2). The number of deaths in males was 56848 (95% UI: 6655–109071) and the number of DALYs was 1359557 (95% UI: 158640–2596453); the number of deaths in females was 41063 (95% UI: 5536–78059) and the number of DALYs was 996309 (95% UI: 135988–1907079) (Supplemental file: Figure 1, and Tables 1 and 2).

An age-stratified analysis of deaths and DALYs for secondhand smoke-related TBLC from an age perspective is shown in Supplementary file Figure 2. In 2021, both the number and ASR of deaths and DALYs increases and then decreases with age. The number of deaths from secondhand smoke-related TBL cancer peaked at age 70–74 years at 15731 (95% UI: 1787–30495); the number of DALYs from secondhand smoke-related TBL cancer peaked at age 65–69 years at 380606 (95% UI: 47383–717297) (Supplemental file: Figure 2, and Tables 1 and 2).

From an SDI analytical framework, high-middle SDI had the highest ASR and number of cases, with the following values: an ASR of 1.96 (95% UI: 0.23–3.67) for deaths, and ASR of 47.2 (95% UI: 5.65–87.54) for DALYs, deaths of 39124 (95% UI: 4613–73341) and 936577 of DALYs (95% UI: 111577–1736627). However, the minimum values of ASR and number of cases were localized to Low SDI, with the following values: deaths 770 (95% UI: 94–1556) and DALYs 22327 (95% UI: 2712–45068); ASR of 0.15 (95% UI: 0.02–0.31) for deaths and 3.96 (95% UI: 0.48–8.01) for DALYs (Supplementary file: Figure 3, and Tables 1 and 2).

In the 54 GBD regions, the number of deaths and DALYs due to secondhand smoke-related TBL cancer deaths in Asia were the highest at 73650 (95% UI: 9146–136835) and 1750750 (95% UI: 220174– 3280225); although Oceania had the lowest number at 51 (95% UI: 7–113) and 1411 (95% UI: 179–3141). Furthermore, the ASR of deaths from secondhand smoke-related TBL cancer and ASR of DALYs were the highest in East Asia with 2.75 (95% UI: 0.34–5.18) and 62.42 (95% UI: 7.79–116.4), respectively, while Western Africa had the lowest with 0.07 (95% UI: 0.01– 0.15) and 1.73 ( 95% UI: 0.21–3.3) (Supplementary file: Figure 4, and Tables 3 and 4).

**Figure 1:**
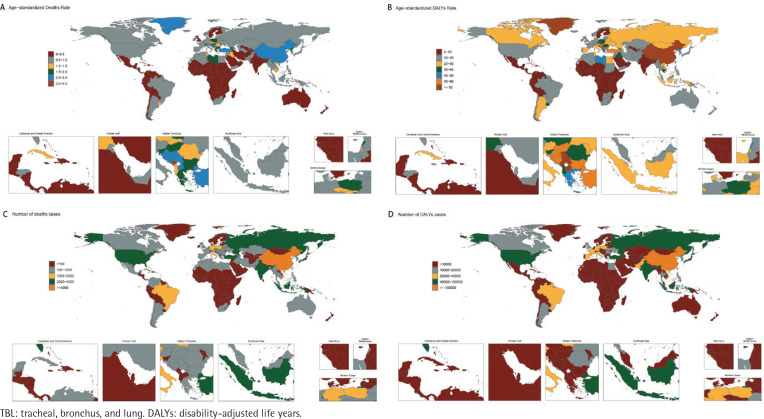
Number and age-standardized rate of secondhand smoke-related TBL cancer-related deaths and DALYs, across countries and territories in 2021: A) Age-standardized deaths rate per 100000; B) Age-standardized DALYs rate per 100000; C) Deaths; D) DALYs

The disease burden of secondhand smoke-related TBL cancer was assessed nationally. In 2021, China had the highest number of deaths and DALYs of secondhand smoke-related TBL cancer, with the following values: deaths 58034 (95% UI: 7170–109625) and DALYs 1359730 (95% UI: 170188–2537368). Antigua and Barbuda, Grenada, Dominica, Cook Islands, Marshall Islands, Palau, Niue, Nauru, Saint Kitts and Nevis, Saint Lucia, Saint Vincent and the Grenadines, San Marino, Sao Tome and Principe, Tokelau and Tuvalu have little to no burden of the disease. Regarding ASR, Montenegro has the highest (Figure 1; and Supplementary file Tables 1 and 2).

### Temporal trend for secondhand smoke-related tracheal, bronchus, and lung cancer burden from 1990 to 2021

**Figure 2:**
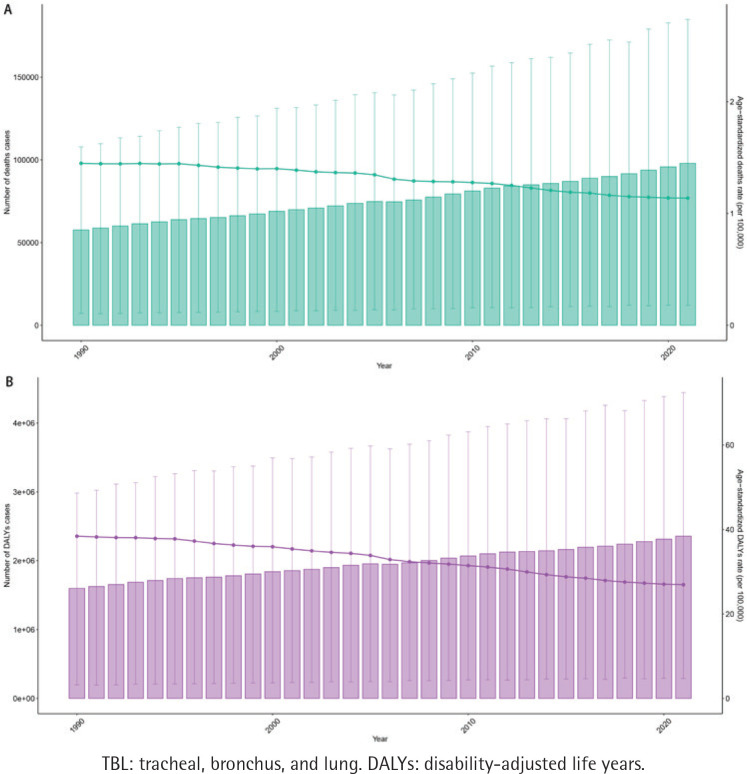
Trends in the numbers and age-standardized rates of secondhand smoke-related TBL cancer-related deaths and DALYs, globally from 1990 to 2021: A) Trends in deaths and age-standardized deaths rate per 100000; B) Trends in DALYs and age-standardized DALYs rate per 100000

We analyzed trends in the burden of disease for secondhand smoke-related TBL cancer from 1990 to 2021. The disease shows an upward trend in both deaths and DALYs. Globally, the number of deaths doubled from 57618 (7083–107842) in 1990 to 97911 (11955–184913) in 2021. The number of DALYs increased from 1598871 (196922-2982788) to 2355866 (290211–4442996). But, ASDR and ASDAR showed a decreasing trend. ASDR reduced from 1.45 (0.18–2.72) in 1990 to 1.14 (0.14–2.15), and ASDAR decreased from 38.4 (4.72–71.68) in 1990 to 26.93 (3.32–50.83) in 2021 (Figure 2, Tables 1 and 2).

From a gender perspective, the analysis of the temporal trends in disease burden shows that the change in the number of deaths and DALYs for both sexes was consistent with the total population, showing an increasing trend. However, the ASR shows a downward trend from 1990 to 2021 (Supplementary file: Figure 5, and Tables 1 and 2). Except for the age groups of 25–29, 35–39 and 40–44 years, the trends in the number of deaths and DALYs is increasing in all age groups. The ASDR and ASDAR are on a downward trend in all age groups except for the age group ≥80 years, which showed an increasing trend in ASR from 1990 to 2021, which was inconsistent with the overall ASR trend (Supplementary file: Figure 6, and Tables 1 and 2). The ASR trends in each SDI were consistent with the overall trend, showing a decreasing trend, and the number of deaths and DALYs in each SDI showed concordance with the overall trend except for High SDI (Supplementary file: Figure 7, and Tables 1 and 2).

**Figure 3:**
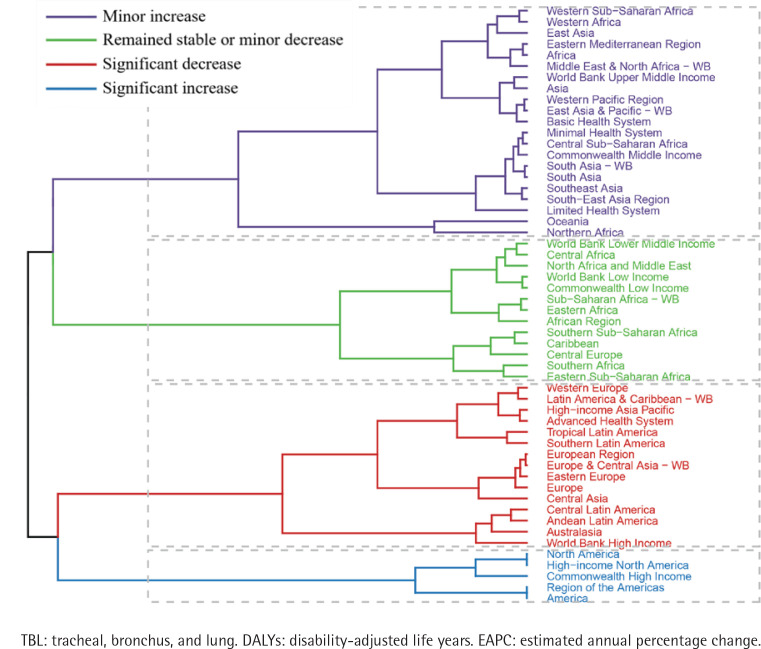
Results of cluster analysis based on the EAPC values of secondhand smoke-related TBL cancer-related age-standardized rates for deaths and DALYs, from 1990 to 2021

So as to better visualize the change in the burden of secondhand smoke-related TBL cancer from 1990 to 2021 in the 56 GBD regions, we performed stratified cluster sampling analysis. Cluster analysis of the EAPC for the ASRs revealed important differences in disease burden trends across regions. As shown in Figure 3, ASR showed a significant increase in five countries including North America, High-income North America, Commonwealth High Income, Region of the Americas and America, and a significant decreasing trend in another 15 regions including: Western Europe, Latin America and Caribbean - WB, High-income Asia Pacific, Advanced Health System, Tropical Latin America, Southern Latin America, European Region, Europe and Central Asia -WB, Eastern Europe, Europe, Central Asia, Central Latin America, Andean Latin America, Australasia, and World Bank High Income (Figure 3, Tables 3 and 4).

**Table 3:** Deaths and the age-standardized deaths rate (ASDR) of secondhand smoke-related TBL at GBD region levels, in 1990 and 2021, and ASDR trends from 1990 to 2021

	*Deaths (95% UI)* *1990*	*Age-standardized* *deaths* *rate/100000* *(95% UI)* *1990*	*Deaths (95% UI)* *2021*	*Age-standardized* *deaths* *rate/100000* *(95% UI)* *2021*	*EAPC (95% CI)*
**GBD region**					
Advanced Health System	28640 (3479–54236)	1.77 (0.21–3.34)	23611 (3023–46038)	0.85 (0.11–1.65)	-2.45 (-2.57 – -2.34)
Africa	870 (109–1649)	0.31 (0.04–0.59)	1997 (251–3851)	0.31 (0.04–0.61)	0.07 (-0.02–0.15)
African Region	488 (62-929)	0.22 (0.03–0.42)	854 (109–1628)	0.17 (0.02–0.33)	-0.93 (-0.99 – -0.86)
America	9257 (1131–17660)	1.53 (0.19–2.91)	7402 (937–14295)	0.55 (0.07–1.05)	-3.5 (-3.61 – -3.38)
Andean Latin America	48 (6–91)	0.24 (0.03–0.45)	67 (8–132)	0.11 (0.01–0.22)	-2.9 (-3.18 – -2.63)
Asia	28835 (3609–53646)	1.48 (0.18–2.75)	73650 (9146–136835)	1.49 (0.18–2.77)	-0.08 (-0.19–0.02)
Australasia	284 (30–604)	1.22 (0.13–2.61)	242 (26–526)	0.47 (0.05–1.01)	-3.04 (-3.14 – -2.95)
Basic Health System	26432 (3284–49469)	1.83 (0.23–3.42)	68619 (8429–127822)	1.87 (0.23–3.49)	0.02 (-0.07–0.11)
Caribbean	269 (34–535)	1.07 (0.13–2.13)	357 (38–727)	0.66 (0.07–1.34)	-1.56 (-1.73 – -1.39)
Central Africa	39 (4–79)	0.14 (0.02–0.28)	73 (7–160)	0.11 (0.01–0.23)	-0.88 (-1.08 – -0.68)
Central Asia	709 (92–1415)	1.45 (0.19–2.9)	600 (78–1192)	0.73 (0.09–1.44)	-1.8 (-1.95 – -1.65)
Central Europe	3808 (480–7293)	2.5 (0.32–4.79)	3494 (438–6729)	1.61 (0.2–3.1)	-1.46 (-1.64 – -1.27)
Central Latin America	338 (42–634)	0.43 (0.05–0.8)	455 (55–866)	0.18 (0.02–0.35)	-3 (-3.12 – -2.89)
Central Sub-Saharan Africa	38 (5–79)	0.16 (0.02–0.34)	84 (10–180)	0.14 (0.02–0.31)	-0.44 (-0.75 – -0.13)
Commonwealth High Income	2870 (360–5430)	1.98 (0.25–3.72)	1482 (190–2936)	0.57 (0.07–1.12)	-4.03 (-4.08 – -3.98)
Commonwealth Low Income	154 (21–323)	0.18 (0.02–0.37)	345 (38–753)	0.15 (0.02–0.33)	-0.72 (-0.92 – -0.52)
Commonwealth Middle Income	1711 (211–3177)	0.28 (0.03–0.52)	4220 (581–8091)	0.27 (0.04–0.52)	-0.46 (-0.64 – -0.29)
East Asia	21359 (2640–40336)	2.6 (0.32–4.9)	59196 (7267–111539)	2.75 (0.34–5.18)	0.14 (0.01–0.26)
East Asia and Pacific - WB	26397 (3289–49231)	2.03 (0.25–3.8)	68210 (8450–127250)	2.07 (0.26–3.85)	-0.03 (-0.14–0.07)
Eastern Africa	126 (15–253)	0.18 (0.02–0.37)	218 (27–438)	0.14 (0.02–0.28)	-1.04 (-1.11 – -0.97)
Eastern Europe	4323 (561–8351)	1.5 (0.19–2.9)	2827 (354–5554)	0.8 (0.1–1.58)	-2.1 (-2.26 – -1.93)
Eastern Mediterranean Region	1211 (150–2229)	0.69 (0.08–1.27)	3059 (364–5984)	0.7 (0.08–1.37)	0.04 (-0.09–0.17)
Eastern Sub-Saharan Africa	91 (11–179)	0.12 (0.01–0.23)	142 (18–272)	0.08 (0.01–0.16)	-1.36 (-1.45 – -1.26)
Europe	18559 (2217–34858)	1.8 (0.22–3.38)	14762 (1881–28407)	0.97 (0.12–1.86)	-2.03 (-2.12 – -1.94)
Europe and Central Asia - WB	18947 (2267–35564)	1.79 (0.21–3.35)	14961 (1910–28907)	0.94 (0.12–1.82)	-2.08 (-2.17 – -1.98)
European Region	19056 (2280–35757)	1.79 (0.21–3.35)	15116 (1930–29190)	0.94 (0.12–1.82)	-2.07 (-2.16 – -1.98)
High-income Asia Pacific	2639 (344–5051)	1.32 (0.17–2.53)	3440 (436–6957)	0.68 (0.09–1.4)	-2.41 (-2.64 – -2.19)
High-income North America	7019 (857–13570)	2.09 (0.25–4.03)	4568 (588–9059)	0.7 (0.09–1.38)	-3.75 (-3.9 – -3.6)
Latin America and Caribbean - WB	2253 (278–4279)	0.86 (0.11–1.63)	2849 (338–5575)	0.4 (0.05–0.79)	-2.58 (-2.66 – -2.49)
Limited Health System	2371 (305–4471)	0.31 (0.04–0.58)	5432 (718–10478)	0.28 (0.04–0.53)	-0.6 (-0.72 – -0.47)
Middle East and North Africa - WB	867 (105–1593)	0.73 (0.09–1.34)	2416 (289–4727)	0.72 (0.09–1.42)	0.13 (0.04–0.21)
Minimal Health System	93 (10–199)	0.16 (0.02–0.34)	166 (18–349)	0.13 (0.01–0.28)	-0.42 (-0.52 – -0.32)
North Africa and Middle East	2151 (237–4062)	1.31 (0.15–2.46)	4534 (526–8819)	1.04 (0.12–2.03)	-0.78 (-0.89 – -0.66)
North America	7017 (857–13567)	2.09 (0.25–4.02)	4567 (588–9057)	0.7 (0.09–1.38)	-3.75 (-3.9 – -3.6)
Northern Africa	409 (50–775)	0.68 (0.08–1.3)	1223 (145–2452)	0.8 (0.09–1.6)	0.74 (0.6–0.88)
Oceania	18 (2–37)	0.67 (0.08–1.41)	51 (7–113)	0.75 (0.1–1.66)	0.3 (0.23–0.38)
Region of the Americas	9257 (1131–17660)	1.53 (0.19–2.91)	7402 (937–14295)	0.55 (0.07–1.05)	-3.5 (-3.61 – -3.38)
South-East Asia Region	2685 (364–5136)	0.39 (0.05–0.76)	6925 (881–13349)	0.39 (0.05–0.75)	-0.36 (-0.49 – -0.23)
South Asia	1481 (186–2775)	0.26 (0.03–0.5)	3869 (526–7497)	0.26 (0.04–0.51)	-0.42 (-0.6 – -0.24)
South Asia - WB	1538 (192–2888)	0.26 (0.03–0.5)	3962 (543–7671)	0.26 (0.04–0.5)	-0.43 (-0.61 – -0.26)
Southeast Asia	2122 (291–3992)	0.86 (0.12–1.63)	5326 (696–10098)	0.83 (0.11–1.57)	-0.33 (-0.42 – -0.24)
Southern Africa	242 (30–473)	0.55 (0.07–1.08)	364 (48–683)	0.38 (0.05–0.71)	-1.3 (-1.5 – -1.09)
Southern Latin America	692 (87–1406)	1.49 (0.19–3.03)	614 (74–1314)	0.71 (0.08–1.52)	-2.31 (-2.53 – -2.09)
Southern Sub-Saharan Africa	223 (27–440)	0.81 (0.1–1.6)	307 (40–584)	0.52 (0.07–0.99)	-1.48 (-1.7 – -1.27)
Sub-Saharan Africa - WB	463 (57–900)	0.21 (0.03–0.41)	777 (96–1494)	0.16 (0.02–0.3)	-1.08 (-1.18 – -0.97)
Tropical Latin America	916 (112–1758)	1.02 (0.12–1.96)	1368 (161–2745)	0.53 (0.06–1.07)	-2.3 (-2.4 – -2.19)
Western Africa	54 (7–105)	0.07 (0.01–0.13)	119 (15–227)	0.07 (0.01–0.14)	-0.01 (-0.11–0.09)
Western Europe	9027 (1054–17210)	1.65 (0.19–3.14)	6227 (775–12307)	0.73 (0.09–1.43)	-2.59 (-2.68 – -2.49)
Western Pacific Region	24526 (3062–46045)	2.19 (0.27–4.11)	63797 (7954–119778)	2.24 (0.28–4.19)	-0.03 (-0.13–0.08)
Western Sub-Saharan Africa	64 (8–125)	0.07 (0.01–0.14)	142 (18–278)	0.07 (0.01–0.15)	-0.05 (-0.13–0.03)
World Bank High Income	22626 (2729–43255)	1.79 (0.22–3.43)	18085 (2290–35374)	0.78 (0.1–1.53)	-2.75 (-2.87 – -2.62)
World Bank Low Income	579 (70–1165)	0.41 (0.05–0.81)	1036 (111–2211)	0.33 (0.04–0.71)	-0.7 (-0.78 – -0.62)
World Bank Lower Middle Income	5406 (721–10104)	0.53 (0.07–0.99)	10749 (1415–20488)	0.43 (0.06–0.82)	-0.84 (-0.93 – -0.75)
World Bank Upper Middle Income	28923 (3551–53430)	1.95 (0.24–3.61)	67958 (8272–127251)	1.94 (0.24–3.63)	-0.08 (-0.17–0.02)

TBL: tracheal, bronchus, and lung.

**Table 4:** DALYs and the age-standardized DALYs rate (ASDAR) of secondhand smoke-related TBL cancer at GBD region levels, in 1990 and 2021, and ASDAR trends from 1990 to 2021

	*DALYs (95% UI)* *1990*	*Age-standardized* *DALYs rate/100000* *(95% UI)* *1990*	*DALYs (95% UI)* *2021*	*Age-standardized* *DALYs rate/100000* *(95% UI)* *2021*	*EAPC (95% CI)*
**GBD region**					
Advanced Health System	784731 (94951–1481506)	49.4 (5.98–93.18)	557158 (71591–1085194)	21.62 (2.78–42.11)	–2.75 (-2.87 – -2.63)
Africa	25171 (3121–47678)	8.12 (1.01–15.39)	56799 (7103–109181)	7.87 (0.99–15.2)	-0.03 (-0.12–0.06)
African Region	14320 (1793–27383)	5.85 (0.74–11.15)	24559 (3163–47109)	4.34 (0.55–8.28)	-1.05 (-1.13 – -0.98)
America	248803 (30435–474899)	41.22 (5.04–78.74)	177646 (22276–343542)	13.32 (1.67–25.78)	-3.8 (-3.91 – -3.69)
Andean Latin America	1323 (168–2503)	6.06 (0.77–11.48)	1726 (201–3400)	2.84 (0.33–5.59)	-3.02 (-3.31 – -2.72)
Asia	801827 (101017–1482781)	36.89 (4.62–68.31)	1750750 (220174–3280225)	33.81 (4.25–63.31)	-0.4 (-0.49 – -0.31)
Australasia	7576 (805–16022)	33.37 (3.56–70.55)	5814 (611–12570)	12.12 (1.27–26.13)	-3.2 (-3.28 – -3.12)
Basic Health System	740660 (92824–1382863)	46.5 (5.8–86.83)	1639957 (203692–3068700)	42.54 (5.28–79.59)	-0.36 (-0.45 – -0.28)
Caribbean	6280 (799–12386)	23.99 (3.05–47.31)	8086 (863–16491)	14.96 (1.6–30.52)	-1.59 (-1.78 – -1.4)
Central Africa	1165 (131–2298)	3.68 (0.42–7.34)	2244 (226–4917)	2.88 (0.29–6.27)	-0.91 (-1.11 – -0.71)
Central Asia	21351 (2754–42678)	41.95 (5.4–83.79)	16793 (2175–33398)	18.75 (2.44–37.2)	-2.24 (-2.36 – -2.12)
Central Europe	110561 (13890–211809)	72.72 (9.13–139.01)	86225 (10669–165607)	42.31 (5.22–81.04)	-1.8 (-2.01 – -1.59)
Central Latin America	8867 (1096–16588)	10.18 (1.26–19.06)	11230 (1346–21277)	4.39 (0.53–8.31)	-3.02 (-3.14 – -2.9)
Central Sub-Saharan Africa	1168 (143–2457)	4.47 (0.54–9.33)	2671 (297–5691)	3.99 (0.45–8.51)	-0.42 (-0.73 – -0.11)
Commonwealth High Income	75637 (9571–142126)	54.61 (6.92–102.13)	35274 (4500–69790)	14.5 (1.84–28.6)	-4.28 (-4.33 – -4.23)
Commonwealth Low Income	4373 (589–9151)	4.68 (0.63–9.75)	9323 (1023–20090)	3.8 (0.42–8.25)	-0.87 (-1.05 – -0.68)
Commonwealth Middle Income	49545 (6130–91846)	7.21 (0.89–13.37)	118347 (16268–226886)	7.03 (0.97–13.46)	-0.41 (-0.58 – -0.24)
East Asia	598410 (74708–1128102)	64.41 (7.99–121.28)	1387975 (172611–2594496)	62.42 (7.79–116.4)	-0.19 (-0.29 – -0.08)
East Asia and Pacific - WB	731133 (91767–1354847)	51.04 (6.38–94.65)	1598992 (200664–3001820)	47.67 (5.99–89.73)	-0.33 (-0.42 – -0.24)
Eastern Africa	3704 (430–7357)	4.79 (0.56–9.61)	6405 (778–12869)	3.53 (0.43–7.1)	-1.17 (-1.26 – -1.09)
Eastern Europe	125653 (16106–243121)	43.66 (5.59–84.28)	75436 (9497–147003)	22.23 (2.83–43.42)	-2.3 (-2.47 – -2.13)
Eastern Mediterranean region	33885 (4180–62336)	17.47 (2.16–32.21)	86064 (10272–168272)	17.26 (2.05–33.77)	-0.03 (-0.17–0.12)
Eastern Sub-Saharan Africa	2737 (336–5391)	3.24 (0.4–6.4)	4306 (533–8218)	2.21 (0.27–4.22)	-1.48 (-1.59 – -1.38)
Europe	520362 (61922–976102)	51.74 (6.17–96.91)	368255 (46999–707535)	25.83 (3.3–49.6)	-2.28 (-2.39 – -2.17)
Europe and Central Asia - WB	532292 (63443–998303)	51.31 (6.12–96.09)	374493 (47904–721946)	25.14 (3.22–48.45)	-2.34 (-2.45 – -2.23)
European region	535230 (63785–1003514)	51.25 (6.11–95.94)	378372 (48414–728993)	25.14 (3.22–48.42)	-2.33 (-2.45 – -2.22)
High-income Asia pacific	65961 (8573–125048)	32 (4.16–60.71)	62673 (7816–128139)	14.83 (1.86–30.46)	-2.74 (-2.97 – -2.51)
High-income North America	188134 (22906-360573)	58.68 (7.12–111.97)	107413 (13713–211496)	17.36 (2.21–34.11)	-4.11 (-4.26 – -3.96)
Latin America and Caribbean - WB	61049 (7622–115829)	21.84 (2.72–41.46)	70552 (8479–137576)	9.84 (1.18–19.2)	-2.73 (-2.82 – -2.63)
Limited health system	68480 (8742–128882)	7.89 (1.01–14.87)	151681 (19893–293701)	7.12 (0.94–13.78)	-0.6 (-0.72 – -0.48)
Middle East and North Africa - WB	24411 (2940–44608)	18.34 (2.22–33.64)	66444 (7990–129697)	17.72 (2.12–34.65)	0.04 (-0.06–0.13)
Minimal Health System	2661 (295–5652)	4.08 (0.45–8.69)	5029 (547–10537)	3.47 (0.38–7.29)	-0.44 (-0.55 – -0.32)
North Africa and Middle East	60176 (6579–114545)	33.1 (3.63–62.82)	120069 (13861–232534)	24.83 (2.88–48.46)	-0.99 (-1.09 – -0.89)
North America	188080 (22897–360466)	58.66 (7.11–111.93)	107381 (13707–211414)	17.35 (2.2–34.09)	-4.11 (-4.26 – -3.96)
Northern Africa	11511 (1408–21791)	17.2 (2.11–32.43)	34040 (4023–68558)	20.07 (2.37–40.29)	0.7 (0.56–0.85)
Oceania	492 (57–1030)	15.9 (1.83–33.37)	1411 (179–3141)	17.75 (2.26–39.22)	0.29 (0.21–0.37)
Region of the Americas	248803 (30435–474899)	41.22 (5.04–78.74)	177646 (22276–343542)	13.32 (1.67–25.78)	-3.8 (-3.91 – -3.69)
South-East Asia Region	77756 (10381–148706)	10.07 (1.36–19.29)	187226 (23689–359492)	9.75 (1.24–18.72)	-0.41 (-0.54 – -0.28)
South Asia	42798 (5403–79876)	6.78 (0.85–12.68)	108253 (14692–210038)	6.85 (0.93–13.28)	-0.35 (-0.52 – -0.17)
South Asia - WB	44402 (5579–82751)	6.83 (0.85–12.78)	110946 (15145–215743)	6.84 (0.94–13.31)	-0.36 (-0.53 – -0.19)
Southeast Asia	59389 (8258–112014)	21.65 (2.99–40.78)	142237 (18519–271986)	20.32 (2.66–38.74)	-0.41 (-0.5 – -0.32)
Southern Africa	7306 (891–14202)	15 (1.82–29.25)	10769 (1435–20112)	10.08 (1.34–18.83)	-1.34 (-1.55 – -1.13)
Southern Latin America	19432 (2494–39639)	41.45 (5.33–84.54)	15436 (1845–33085)	18.3 (2.18–39.2)	-2.57 (-2.81 – -2.33)
Southern Sub-Saharan Africa	6711 (821–13291)	22.55 (2.75–44.64)	8950 (1160–17074)	14.07 (1.82–26.85)	-1.56 (-1.78 – -1.35)
Sub-Saharan Africa - WB	13722 (1681–26786)	5.58 (0.69–10.87)	22825 (2833–43800)	4.04 (0.5–7.77)	-1.15 (-1.26 – -1.05)
Tropical Latin America	25378 (3109–48617)	26.02 (3.18–49.85)	34368 (4093–69074)	13.08 (1.56–26.27)	-2.44 (-2.53 – -2.34)
Western Africa	1485 (187–2899)	1.74 (0.22–3.42)	3342 (410–6434)	1.73 (0.21–3.3)	-0.11 (-0.21 – -0.01)
Western Europe	244694 (28689–466150)	46.96 (5.53–89.32)	150799 (19175–295049)	19.46 (2.49–38.01)	-2.79 (-2.91 – -2.67)
Western Pacific Region	678145 (85409–1268132)	55.28 (6.93–103.34)	1485064 (187692–2776392)	51.92 (6.59–97.34)	-0.31 (-0.41 – -0.22)
Western Sub-Saharan Africa	1781 (227–3460)	1.89 (0.24–3.68)	3995 (492–7784)	1.85 (0.23–3.61)	-0.15 (-0.23 – -0.06)
World Bank High Income	611336 (73515–1163660)	50 (6.01–95.08)	413945 (52602–807081)	19.68 (2.5–38.2)	-3.07 (-3.21 – -2.93)
World Bank Low Income	16917 (2037–33593)	10.47 (1.27–20.92)	29345 (3185–61974)	8.23 (0.88–17.51)	-0.85 (-0.93 – -0.77)
World Bank Lower Middle Income	153618 (20200–288712)	13.64 (1.81–25.55)	297305 (39364–562169)	11.01 (1.45–20.88)	-0.86 (-0.95 – -0.77)
World Bank Upper Middle Income	814655 (100857–1497907)	50.64 (6.24–93.28)	1613222 (197770–3030652)	44.92 (5.52–84.43)	-0.48 (-0.57 – -0.4)

TBL: tracheal, bronchus, and lung. DALYs: disability-adjusted life years.

**Figure 4:**
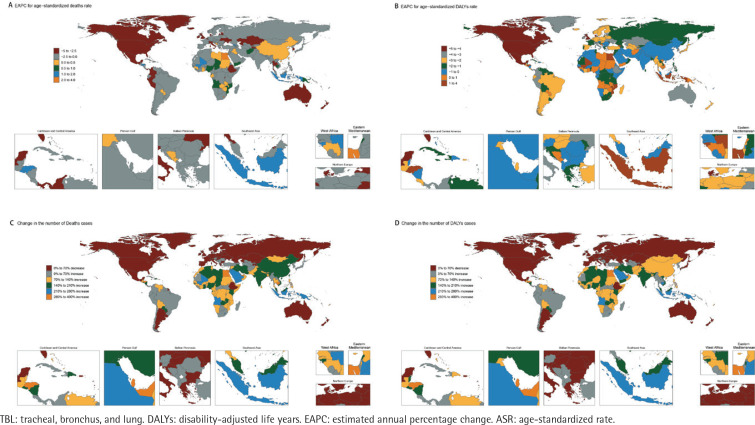
The EAPC of secondhand smoke-related TBL cancer-related ASR and relative change in deaths and DALYs, between 1990 and 2021: A) The EAPC of age-standardized deaths rate; B) The EAPC of age-standardized DALYs rate; C) The change in the number of deaths; D) The change in the number of DALYs

The trend of disease burden varies among countries. Lesotho had the highest increase in ASR (EAPC=3.64; 95% CI: 3.22–4.07) and Mexico had the highest decrease in ASR (EAPC= -4.63; 95% CI: -4.83 – -4.42). Similarly, Lesotho had the highest growth rate in ASDR (EAPC=3.76; 95% CI: 3.28–4.25) and United Kingdom had the most significant decline in ASDR (EAPC= -4.79; 95% CI: -4.86 – -4.73) (Figure 4; and Supplementary file Tables 1 and 2).

## DISCUSSION

This study provides a systematic and comprehensive description of the global burden of tracheal, bronchus, and lung (TBL) cancers attributable to secondhand smoke, utilizing the Global Burden of Disease (GBD) 2021 dataset, and quantifies both disease burden and its share for different SDI levels, geographical regions, sexes, and age groups, over a 30-year period from 1990 to 2021.

In this analysis, we found that while the ASRs of TBL cancer attributable to secondhand smoke have decreased at the global level from 1990 to 2021, the absolute numbers of deaths and DALYs have increased, indicating TBL cancer remains a major cause of the global cancer burden. The expansion of the global population tends to increase the absolute number of cancer cases and deaths, thereby placing greater strain on healthcare systems^4,19,20^. Notably, The World Health Organization (WHO) estimates that by 2050, the global population aged ≥60 years is projected to double. In addition, over the past few decades, numerous effective tobacco control programs and policies have significantly reduced the prevalence of tobacco use, including tobacco taxation, smoking bans in public places, and the WHO Framework Convention on Tobacco Control^21-23^. Despite the fact that the global smoking rate has declined, the absolute number of smokers is still increasing due to population growth. This phenomenon also indicates that the number of deaths and DALYs attributable to TBL cancer due to secondhand smoke is on the rise, yet the ASRs trend downward, which is consistent with previous studies^24^.

Gender differences in work and lifestyle habits are also significant factors influencing mortality. Our study suggests that the number of deaths and DALYs of secondhand smoke-related TBL cancer in men was approximately 1.4 times higher than that of women in 2021. The greater burden of TBL cancer among males may be associated with higher exposure to occupational and environmental carcinogens^25^.

Consistent with prior findings, our age-stratified analysis of deaths and DALYs from secondhand smoke-related TBL cancer in 2021 showed that the burden of disease increased significantly with age^24^, with the highest number of deaths clustered in the age group of 70–74 years and DALYs clustered in the age group 65–69 years. Notably, the ASR showed a declining trend across all age groups except for the ≥80 years group, suggesting that elderly population is more susceptible to immune function decline, thereby further increasing the risk of cancer mortality^26^. These findings highlight that secondhand smoke control could significantly reduce the TBL cancer burden, especially among the elderly. This suggests that targeted interventions addressing demographic shifts are needed, particularly in rapidly aging societies.

This study further examined the burden of secondhand smoke-related TBL cancer across different SDI regions. Similar to previous findings^24^, the ASRs in five SDI regions showed great variation, with the highest number and ASRs of deaths and DALYs in High-middle SDI regions and the lowest number in low SDI regions in 2021. From 1990 to 2021, all SDI regions showed a decreasing trend. The number of deaths and DALYs of High SDI regions have decreased significantly, as smoking is more prevalent in higher SDI levels^27^. These findings suggest that enhanced tobacco control measures coupled with early detection and treatment interventions could effectively mitigate disease burden in high-SDI settings. The remaining SDI regions declined slowly, suggesting that these areas are experiencing the pre-variation phase of disease burden in High SDI areas due to horizontal developmental constraints. All these results reveal a complex relationship between socioeconomic factors and disease burden.

The findings also analyzed the disease burden of secondhand smoke-related TBL cancer at the level of geographical differences. Between 1990 and 2021, the ASRs decreased in most areas, but increased in North America, High-income North America, Commonwealth High Income and Region of the Americas. This difference approximately attributed to the tobacco control measures and the timeline of smoking prevalence across regions^22,23^. Hierarchical cluster analysis also classified each GBD region based on whether ASRs were rising or declining, significant or non-significant, enabling targeted intervention strategies tailored to each region’s epidemiological profile.

At the country level, between 1990 and 2021, Lesotho showed the most significant increase in disease burden, whereas the largest decrease was in ASR in Mexico and ASDR in United Kingdom. China had the largest number of TBL cancer deaths and DALYs attributable to secondhand smoke in 2021. The different burden in secondhand smoke-related TBL cancer among countries likely are reflected by tobacco control policies, sociocultural aspects, economic development, healthcare service levels and medical technology adoption^28-30^.

### Limitations

The limitations of this study are comparable to those of other GBD studies^12^. First of all, since the raw data were based on the GBD database acquired from civil registration, hospital records and vital statistics, the residual confounding factors cannot be avoided. Second, changes in smoking exposure will have a delayed effect on TBL cancer mortality. Therefore, the medium- to long-term success of policies such as tobacco control legislation may not be reflected in the current burden data. Last, our projections consider secondhand smoke alone, while many other risk factors that may significantly increase the burden of TBL cancer are excluded, and this overly simplified model may weaken the accuracy and robustness of our projections.

## CONCLUSIONS

Despite the decline in the burden of secondhand smoke-related TBL cancer over the past 30 years, it remains a significant global health risk and challenge. The findings of this study can serve as a basis for formulating targeted tobacco control policies, which could significantly contribute to reducing the global burden of TBL cancer attributable to SHS.

## Supplementary Material



## Data Availability

All data generated or analyzed in this study are included in this article and in the Supplementary file.
