# Supplementary material for: The global, regional, and national burden of secondhand smoke-related tracheal, bronchus, and lung cancer: Results from the Global Burden of Disease study 2021
Source: Tob Induc Dis. 2025 Jun 12;23:10.18332/tid/205049. doi: 10.18332/tid/205049 (PMC12160775; doi:10.18332/tid/205049)
Supplement: Supplementary file 1 [file TID-23-80-s1.pdf]

Supplemental Table 1. The number of deaths cases and the age-standardized deaths rate of secondhand smoke-related TBL cancer at country levels in 1990 and 2021, and its trends from 1990 to 2021. Abbreviations: TBL cancer, tracheal, bronchus, and lung cancer.

	Number of deaths cases (95% UI) in 1990	The age-standardized deaths rate/100 000 (95% UI) in 1990	Number of deaths cases (95% UI) in 2021	The age-standardized deaths rate/100 000 (95% UI) in 2021	EAPC (95% CI)
Country					
Afghanistan	30 (3-82)	0.44 (0.04-1.16)	46 (4-111)	0.47 (0.05-1.1)	0.64 (0.48/0.79)
Albania	40 (5-80)	1.98 (0.24-3.93)	65 (7-134)	1.48 (0.16-3.06)	-0.86 (-1.13/-0.59)
Algeria	70 (9-139)	0.63 (0.08-1.24)	176 (22-354)	0.52 (0.07-1.06)	-0.33 (-0.57/-0.08)
American Samoa	0 (0-1)	1.39 (0.14-3.42)	1 (0-1)	1.27 (0.12-3.12)	-0.24 (-0.3/-0.19)
Andorra	1 (0-3)	1.93 (0.22-4.59)	1 (0-3)	0.8 (0.09-1.77)	-2.65 (-2.88/-2.42)
Angola	9 (1-22)	0.21 (0.03-0.5)	32 (4-71)	0.25 (0.03-0.55)	0.84 (0.49/1.19)
Antigua and Barbuda	0 (0-0)	0.31 (0.04-0.68)	0 (0-1)	0.21 (0.02-0.49)	-1.32 (-1.6/-1.04)
Argentina	524 (60-1124)	1.62 (0.19-3.46)	423 (53-989)	0.77 (0.1-1.79)	-2.22 (-2.5/-1.94)
Armenia	68 (8-134)	2.36 (0.27-4.67)	76 (10-145)	1.73 (0.23-3.28)	-0.74 (-1/-0.47)
Australia	240	1.24	205	0.47	-3.07

	(23-517)	(0.12-2.65)	(21-453)	(0.05-1.03)	(-3.19/-2.95)
Austria	131 (14-279)	1.19 (0.13-2.54)	159 (22-311)	0.95 (0.13-1.85)	-0.45 (-0.76/-0.14)
Azerbaijan	78 (10-156)	1.47 (0.18-2.97)	110 (14-221)	1.03 (0.13-2.03)	-0.71 (-1/-0.42)
Bahamas	1 (0-2)	0.45 (0.05-0.99)	2 (0-3)	0.37 (0.04-0.84)	-0.23 (-0.37/-0.09)
Bahrain	3 (0-7)	2.25 (0.28-4.44)	9 (1-18)	1.23 (0.15-2.47)	-2.44 (-2.74/-2.14)
Bangladesh	118 (15-263)	0.25 (0.03-0.56)	273 (29-632)	0.2 (0.02-0.46)	-1.03 (-1.28/-0.79)
Barbados	1 (0-1)	0.18 (0.02-0.41)	1 (0-1)	0.1 (0.01-0.25)	-2.12 (-2.41/-1.83)
Belarus	162 (16-340)	1.23 (0.12-2.55)	118 (12-249)	0.73 (0.07-1.55)	-2.17 (-2.39/-1.95)
Belgium	334 (43-667)	2.28 (0.29-4.51)	204 (24-423)	0.94 (0.11-1.94)	-2.6 (-2.81/-2.39)
Belize	0 (0-1)	0.24 (0.03-0.58)	1 (0-1)	0.17 (0.02-0.39)	-0.97 (-1.29/-0.66)
Benin	2 (0-5)	0.1 (0.01-0.23)	3 (0-8)	0.07 (0.01-0.16)	-1.34 (-1.4/-1.27)
Bermuda	1 (0-2)	1.1 (0.1-2.56)	1 (0-2)	0.65 (0.05-1.49)	-1.48 (-1.64/-1.32)
Bhutan	0 (0-1)	0.19 (0.02-0.44)	1 (0-3)	0.18 (0.02-0.48)	-0.26 (-0.44/-0.07)
Bolivia (Plurinational State of)	9 (1-18)	0.28 (0.03-0.57)	17 (2-37)	0.19 (0.02-0.42)	-0.99 (-1.29/-0.68)
Bosnia and Herzegovina	95 (10-186)	2.16 (0.23-4.24)	139 (18-287)	2.22 (0.28-4.57)	0.37 (0.04/0.71)

Botswana	4 (0-9)	0.68 (0.07-1.58)	6 (1-15)	0.43 (0.05-0.99)	-1.89 (-2.26/-1.52)
Brazil	907 (110-1742)	1.03 (0.13-1.99)	1342 (159-2687)	0.53 (0.06-1.07)	-2.34 (-2.45/-2.24)
Brunei Darussalam	2 (0-4)	1.95 (0.26-3.82)	3 (0-6)	0.78 (0.07-1.71)	-2.53 (-2.78/-2.28)
Bulgaria	264 (32-499)	2.12 (0.26-4.01)	232 (25-499)	1.76 (0.19-3.8)	-0.26 (-0.45/-0.06)
Burkina Faso	5 (1-10)	0.11 (0.01-0.24)	10 (1-22)	0.11 (0.01-0.25)	-0.06 (-0.3/0.17)
Burundi	2 (0-5)	0.1 (0.01-0.22)	3 (0-6)	0.05 (0.01-0.11)	-2.65 (-3.05/-2.25)
Cabo Verde	1 (0-1)	0.23 (0.02-0.55)	1 (0-3)	0.28 (0.03-0.64)	0.24 (-0.18/0.66)
Cambodia	39 (4-88)	0.9 (0.08-2.03)	149 (14-311)	1.25 (0.12-2.62)	0.97 (0.82/1.11)
Cameroon	6 (1-14)	0.13 (0.01-0.32)	13 (1-31)	0.11 (0.01-0.25)	-0.78 (-0.88/-0.69)
Canada	842 (90-1598)	2.65 (0.28-5.02)	581 (57-1186)	0.81 (0.08-1.66)	-3.8 (-3.94/-3.67)
Central African Republic	2 (0-6)	0.19 (0.02-0.48)	3 (0-10)	0.14 (0.01-0.37)	-1.21 (-1.39/-1.03)
Chad	2 (0-5)	0.08 (0.01-0.19)	6 (1-14)	0.1 (0.01-0.25)	0.93 (0.87/0.99)
Chile	92 (10-201)	0.93 (0.1-2.02)	126 (15-269)	0.48 (0.06-1.04)	-2.02 (-2.09/-1.94)
China	20758 (2564-39187)	2.63 (0.32-4.96)	58034 (7170-109625)	2.8 (0.35-5.27)	0.16 (0.03/0.28)
Colombia	73 (8-149)	0.43 (0.05-0.	100 (11-209)	0.18 (0.02-0.	-3.18 (-3.32/-3.

		88)		38)	03)
Comoros	0 (0-1)	0.2 (0.02-0.44)	1 (0-2)	0.17 (0.02-0.4)	-0.73 (-0.84/-0.63)
Congo	3 (0-8)	0.3 (0.04-0.72)	7 (1-17)	0.24 (0.02-0.59)	-1.11 (-1.4/-0.83)
Cook Islands	0 (0-0)	1.55 (0.2-3.52)	0 (0-1)	1.16 (0.13-2.85)	-1.17 (-1.31/-1.02)
Costa Rica	7 (1-17)	0.4 (0.04-0.99)	11 (1-27)	0.19 (0.02-0.48)	-2.29 (-2.45/-2.13)
C?te d'Ivoire	6 (1-15)	0.16 (0.02-0.38)	12 (1-31)	0.11 (0.01-0.29)	-2.4 (-3.11/-1.68)
Croatia	226 (31-426)	3.56 (0.49-6.72)	191 (23-400)	2.19 (0.26-4.59)	-1.06 (-1.23/-0.89)
Cuba	207 (25-427)	2.03 (0.25-4.18)	264 (28-576)	1.32 (0.14-2.88)	-1.42 (-1.62/-1.21)
Cyprus	9 (1-18)	1.15 (0.12-2.31)	18 (2-36)	0.88 (0.11-1.76)	-0.26 (-0.5/-0.02)
Czechia	369 (41-753)	2.74 (0.31-5.58)	274 (31-554)	1.27 (0.15-2.57)	-2.38 (-2.46/-2.31)
Democratic People's Republic of Korea	306 (36-621)	1.92 (0.23-3.9)	506 (39-1185)	1.52 (0.12-3.56)	-0.75 (-0.85/-0.65)
Democratic Republic of the Congo	21 (2-45)	0.13 (0.01-0.29)	37 (4-89)	0.1 (0.01-0.24)	-1.18 (-1.53/-0.84)
Denmark	209 (23-430)	2.77 (0.31-5.71)	99 (12-206)	0.85 (0.1-1.76)	-3.99 (-4.09/-3.89)
Djibouti	0 (0-1)	0.27 (0.03-0.62)	2 (0-3)	0.28 (0.03-0.58)	0.16 (0.12/0.2)
Dominica	0 (0-1)	0.38 (0.04-0.94)	0 (0-1)	0.3 (0.03-0.75)	-0.79 (-1.05/-0.53)
Dominican Republic	14 (1-31)	0.41	34 (3-87)	0.35	-0.24

		(0.04-0.89)		(0.03-0.87)	(-0.58/0.1)
Ecuador	10 (1-21)	0.19 (0.02-0.41)	16 (2-31)	0.1 (0.01-0.2)	-2.16 (-2.4/-1.92)
Egypt	115 (12-222)	0.4 (0.04-0.77)	536 (59-1118)	0.86 (0.09-1.81)	3.41 (2.88/3.93)
El Salvador	4 (0-9)	0.12 (0.01-0.29)	11 (1-25)	0.17 (0.02-0.41)	1.18 (0.95/1.42)
Equatorial Guinea	1 (0-1)	0.28 (0.03-0.67)	2 (0-4)	0.33 (0.04-0.73)	0.72 (0.59/0.86)
Eritrea	2 (0-6)	0.17 (0.02-0.46)	5 (0-14)	0.17 (0.02-0.44)	-0.1 (-0.27/0.07)
Estonia	40 (4-84)	1.94 (0.21-4.06)	23 (3-49)	0.87 (0.1-1.88)	-2.63 (-2.89/-2.37)
Eswatini	1 (0-2)	0.35 (0.04-0.78)	2 (0-4)	0.33 (0.04-0.72)	-0.07 (-0.63/0.49)
Ethiopia	33 (4-69)	0.15 (0.02-0.31)	32 (4-63)	0.07 (0.01-0.14)	-2.82 (-3.06/-2.57)
Fiji	1 (0-2)	0.35 (0.04-0.65)	2 (0-4)	0.29 (0.03-0.6)	-0.74 (-1.04/-0.43)
Finland	53 (6-119)	0.77 (0.08-1.72)	39 (4-91)	0.34 (0.03-0.77)	-2.27 (-2.45/-2.1)
France	839 (83-1930)	1.13 (0.11-2.59)	764 (80-1787)	0.65 (0.07-1.5)	-1.71 (-1.99/-1.43)
Gabon	2 (0-3)	0.26 (0.03-0.54)	3 (0-7)	0.27 (0.03-0.61)	0.04 (-0.06/0.14)
Gambia	0 (0-1)	0.11 (0.01-0.26)	1 (0-2)	0.09 (0.01-0.23)	-0.95 (-1.2/-0.7)
Georgia	107 (13-219)	1.66 (0.2-3.38)	102 (13-201)	1.73 (0.21-3.39)	1.62 (1.14/2.1)

Germany	1581 (184-3204)	1.34 (0.16-2.7)	1219 (124-2624)	0.71 (0.07-1.52)	-1.98 (-2.04/-1.93)
Ghana	3 (0-7)	0.05 (0.01-0.1)	9 (1-21)	0.06 (0.01-0.13)	0.45 (0.33/0.57)
Greece	392 (50-762)	2.58 (0.33-5.03)	373 (34-754)	1.7 (0.16-3.42)	-1.42 (-1.59/-1.25)
Greenland	2 (0-5)	5.99 (0.57-13.07)	2 (0-4)	2.35 (0.24-5.62)	-3.21 (-3.39/-3.03)
Grenada	0 (0-0)	0.28 (0.03-0.7)	0 (0-0)	0.16 (0.02-0.36)	-1.78 (-2.16/-1.4)
Guam	1 (0-2)	1.52 (0.18-3.49)	2 (0-5)	0.98 (0.09-2.48)	-0.99 (-1.26/-0.72)
Guatemala	5 (1-9)	0.15 (0.02-0.28)	10 (1-19)	0.09 (0.01-0.17)	-2.12 (-2.42/-1.81)
Guinea	5 (1-13)	0.16 (0.02-0.42)	11 (1-27)	0.19 (0.02-0.5)	0.49 (0.4/0.57)
Guinea-Bissau	1 (0-1)	0.13 (0.01-0.32)	1 (0-3)	0.18 (0.01-0.44)	1.72 (1.28/2.16)
Guyana	1 (0-2)	0.22 (0.02-0.53)	1 (0-2)	0.14 (0.01-0.34)	-1.49 (-1.69/-1.3)
Haiti	6 (1-14)	0.2 (0.02-0.44)	8 (1-17)	0.11 (0.01-0.25)	-1.72 (-1.98/-1.45)
Honduras	9 (1-22)	0.45 (0.05-1.09)	42 (4-113)	0.68 (0.07-1.83)	1.57 (1.4/1.74)
Hungary	444 (59-847)	3.11 (0.41-5.96)	337 (35-707)	1.86 (0.19-3.87)	-1.83 (-2.12/-1.54)
Iceland	4 (0-8)	1.48 (0.17-2.85)	4 (0-7)	0.65 (0.08-1.31)	-2.6 (-2.69/-2.5)
India	950 (124-1851	0.21 (0.03-0.	2816 (390-5584	0.24 (0.03-0.	0 (-0.2/0.2)

	)	4)	)	47)	
Indonesia	596 (67-1232)	0.62 (0.07-1.29)	2202 (272-4463)	0.97 (0.12-1.98)	1.46 (1.36/1.57)
Iran (Islamic Republic of)	115 (14-231)	0.45 (0.05-0.89)	308 (41-609)	0.4 (0.05-0.8)	-0.19 (-0.39/0.02)
Iraq	103 (12-193)	1.35 (0.16-2.52)	315 (34-639)	1.44 (0.15-2.94)	0.01 (-0.15/0.18)
Ireland	87 (10-183)	2.13 (0.25-4.47)	51 (6-112)	0.65 (0.07-1.44)	-3.82 (-4.03/-3.61)
Israel	53 (7-109)	1.14 (0.14-2.34)	65 (7-150)	0.55 (0.06-1.26)	-2.22 (-2.55/-1.88)
Italy	1827 (210-3455)	2.11 (0.24-4)	1182 (147-2356)	0.86 (0.11-1.69)	-2.94 (-3.01/-2.87)
Jamaica	11 (1-23)	0.63 (0.06-1.31)	13 (1-30)	0.43 (0.05-0.96)	-1.75 (-2.15/-1.35)
Japan	2119 (281-4121)	1.25 (0.17-2.43)	2573 (324-5225)	0.65 (0.08-1.3)	-2.34 (-2.55/-2.13)
Jordan	15 (2-28)	1.13 (0.14-2.12)	50 (6-107)	0.67 (0.08-1.44)	-1.64 (-1.92/-1.37)
Kazakhstan	268 (30-588)	2.03 (0.23-4.47)	122 (13-265)	0.65 (0.07-1.41)	-3.52 (-3.72/-3.32)
Kenya	3 (0-7)	0.04 (0-0.09)	10 (1-20)	0.04 (0.01-0.09)	-0.1 (-0.35/0.15)
Kiribati	0 (0-1)	0.73 (0.09-1.62)	1 (0-2)	1.09 (0.14-2.58)	1.17 (0.83/1.52)
Kuwait	6 (1-11)	1.03 (0.13-1.96)	14 (2-28)	0.54 (0.07-1.03)	-1.68 (-2.01/-1.36)
Kyrgyzstan	46 (5-92)	1.5 (0.18-3)	36 (5-73)	0.75 (0.09-1.48)	-1.49 (-1.91/-1.07)
Lao People's	24 (3-61)	1.19	37 (4-90)	0.86	-1.25

Democratic Republic		(0.13-3)		(0.09-2.08)	(-1.33/-1.17)
Latvia	67 (6-145)	1.85 (0.17-4)	29 (3-67)	0.76 (0.08-1.77)	-2.87 (-3.09/-2.66)
Lebanon	38 (5-76)	1.77 (0.25-3.58)	106 (11-211)	1.75 (0.17-3.46)	0.64 (0.26/1.02)
Lesotho	4 (0-10)	0.48 (0.04-1.16)	13 (1-29)	1.13 (0.09-2.58)	3.64 (3.22/4.07)
Liberia	1 (0-2)	0.08 (0.01-0.16)	1 (0-3)	0.06 (0.01-0.13)	-0.57 (-0.79/-0.35)
Libya	35 (4-68)	1.89 (0.25-3.73)	101 (12-210)	1.98 (0.24-4.11)	0.41 (0.15/0.67)
Lithuania	62 (6-136)	1.35 (0.13-2.96)	38 (4-85)	0.69 (0.06-1.53)	-2.39 (-2.52/-2.26)
Luxembourg	10 (1-21)	1.91 (0.17-3.95)	9 (1-19)	0.85 (0.07-1.83)	-2.54 (-2.66/-2.41)
Madagascar	8 (1-16)	0.16 (0.02-0.31)	10 (1-20)	0.08 (0.01-0.17)	-2.27 (-2.49/-2.04)
Malawi	1 (0-3)	0.03 (0-0.07)	3 (0-8)	0.04 (0-0.1)	0.4 (0.1/0.71)
Malaysia	77 (9-177)	0.86 (0.1-1.97)	213 (20-525)	0.76 (0.07-1.89)	-0.45 (-0.87/-0.03)
Maldives	1 (0-2)	0.98 (0.1-2.15)	1 (0-3)	0.4 (0.04-0.91)	-3.55 (-3.75/-3.34)
Mali	3 (0-7)	0.09 (0.01-0.18)	11 (1-24)	0.12 (0.01-0.28)	1.44 (1.36/1.51)
Malta	5 (1-11)	1.27 (0.16-2.5)	6 (1-12)	0.63 (0.07-1.33)	-2.18 (-2.31/-2.05)
Marshall Islands	0 (0-0)	1.12 (0.1-2.72)	0 (0-1)	1.23 (0.11-3.2)	0.29 (0.21/0.37)
Mauritania	3 (0-7)	0.28	5 (1-10)	0.22	-0.97

		(0.04-0.68)		(0.03-0.49)	(-1.16/-0.78)
Mauritius	5 (1-10)	0.65 (0.07-1.5)	9 (1-21)	0.5 (0.05-1.13)	-1.03 (-1.29/-0.76)
Mexico	174 (22-326)	0.44 (0.06-0.82)	152 (20-291)	0.12 (0.02-0.24)	-4.63 (-4.83/-4.42)
Micronesia (Federated States of)	1 (0-1)	1.17 (0.13-2.44)	1 (0-2)	1.31 (0.15-2.74)	0.37 (0.34/0.4)
Monaco	1 (0-3)	2.22 (0.23-4.84)	2 (0-4)	2.01 (0.2-4.41)	-0.21 (-0.53/0.1)
Mongolia	11 (1-27)	1.08 (0.14-2.61)	21 (2-50)	0.93 (0.11-2.19)	-1 (-1.2/-0.8)
Montenegro	20 (3-39)	3.19 (0.4-6.17)	34 (4-70)	3.45 (0.38-7.06)	0.39 (0.11/0.66)
Morocco	114 (13-223)	0.82 (0.1-1.6)	262 (31-565)	0.77 (0.09-1.66)	-0.34 (-0.53/-0.15)
Mozambique	5 (1-9)	0.08 (0.01-0.16)	10 (1-20)	0.09 (0.01-0.19)	0.9 (0.74/1.05)
Myanmar	222 (24-510)	0.98 (0.11-2.25)	243 (29-477)	0.51 (0.06-1.02)	-2.11 (-2.25/-1.98)
Namibia	1 (0-2)	0.15 (0.02-0.3)	2 (0-4)	0.14 (0.02-0.27)	-0.62 (-0.92/-0.33)
Nauru	0 (0-0)	2.06 (0.26-4.57)	0 (0-0)	1.8 (0.21-3.67)	-0.54 (-0.62/-0.45)
Nepal	16 (2-34)	0.17 (0.02-0.37)	34 (4-70)	0.15 (0.02-0.31)	-0.51 (-0.73/-0.29)
Netherlands	315 (34-680)	1.65 (0.18-3.54)	242 (28-535)	0.72 (0.08-1.59)	-2.14 (-2.36/-1.92)
New Zealand	44 (5-101)	1.15 (0.13-2.63)	37 (4-87)	0.45 (0.05-1.07)	-2.91 (-3.04/-2.79)

Nicaragua	3 (0-6)	0.18 (0.02-0.4)	7 (1-18)	0.15 (0.01-0.37)	-0.29 (-0.45/-0.14)
Niger	2 (0-4)	0.07 (0.01-0.16)	4 (0-10)	0.06 (0.01-0.12)	-0.6 (-0.81/-0.4)
Nigeria	7 (1-14)	0.02 (0-0.03)	17 (2-34)	0.02 (0-0.04)	0.84 (0.69/0.99)
Niue	0 (0-0)	1.05 (0.11-2.51)	0 (0-0)	1.24 (0.14-3.24)	0.45 (0.36/0.55)
North Macedonia	52 (7-103)	2.74 (0.35-5.38)	86 (10-179)	2.55 (0.29-5.31)	-0.25 (-0.63/0.13)
Northern Mariana Islands	0 (0-1)	1.93 (0.23-4.41)	1 (0-2)	1.51 (0.17-3.54)	-0.96 (-1.07/-0.84)
Norway	58 (7-110)	0.94 (0.12-1.78)	50 (6-101)	0.51 (0.07-1.03)	-2.13 (-2.43/-1.84)
Oman	2 (0-4)	0.27 (0.03-0.65)	4 (0-8)	0.2 (0.02-0.47)	-0.8 (-1.16/-0.45)
Pakistan	397 (45-814)	0.72 (0.08-1.48)	744 (81-1612)	0.62 (0.07-1.34)	-0.78 (-1.15/-0.4)
Palau	0 (0-0)	1.77 (0.2-4.54)	0 (0-1)	1.72 (0.17-4.27)	-0.11 (-0.18/-0.04)
Palestine	12 (1-23)	1.4 (0.18-2.72)	27 (3-56)	1.1 (0.12-2.27)	-0.96 (-1.15/-0.77)
Panama	6 (1-14)	0.4 (0.04-0.93)	7 (1-17)	0.15 (0.02-0.38)	-3.39 (-3.55/-3.23)
Papua New Guinea	10 (1-22)	0.61 (0.06-1.35)	34 (4-80)	0.76 (0.09-1.81)	0.68 (0.62/0.73)
Paraguay	9 (1-22)	0.42 (0.04-1.03)	27 (3-68)	0.47 (0.05-1.19)	0.43 (0.29/0.57)
Peru	29 (3-54)	0.25 (0.03-0.	34 (4-70)	0.1 (0.01-0.	-3.89 (-4.34/-3.

		46)		21)	43)
Philippines	309 (41-578)	1.15 (0.15-2.17)	579 (70-1105)	0.72 (0.09-1.37)	-1.58 (-1.72/-1.43)
Poland	1319 (175-2499)	2.99 (0.39-5.69)	1082 (161-2271)	1.5 (0.22-3.14)	-2.48 (-2.67/-2.28)
Portugal	132 (12-274)	0.97 (0.09-2.02)	128 (11-283)	0.58 (0.05-1.29)	/-1.74 (-1.96/-1.52)
Puerto Rico	12 (1-28)	0.34 (0.04-0.77)	12 (1-26)	0.16 (0.01-0.37)	-2.3 (-2.42/-2.17)
Qatar	2 (0-3)	1.68 (0.22-3.37)	7 (1-13)	0.86 (0.11-1.74)	-2.23 (-2.81/-1.64)
Republic of Korea	493 (59-973)	1.67 (0.2-3.29)	834 (91-1966)	0.88 (0.1-2.07)	-2.75 (-3.16/-2.34)
Republic of Moldova	63 (8-122)	1.36 (0.16-2.64)	44 (5-88)	0.74 (0.09-1.47)	-1.46 (-1.75/-1.17)
Romania	398 (43-825)	1.38 (0.15-2.84)	443 (34-1010)	1.29 (0.1-2.95)	-0.42 (-0.65/-0.19)
Russian Federation	2421 (289-4630)	1.29 (0.15-2.46)	2040 (237-4063)	0.85 (0.1-1.68)	-1.41 (-1.66/-1.15)
Rwanda	5 (1-13)	0.16 (0.02-0.43)	8 (1-24)	0.13 (0.01-0.36)	-1.36 (-1.74/-0.98)
Saint Kitts and Nevis	0 (0-0)	0.3 (0.04-0.66)	0 (0-0)	0.17 (0.02-0.41)	-1.81 (-1.94/-1.68)
Saint Lucia	0 (0-1)	0.36 (0.04-0.84)	0 (0-1)	0.14 (0.01-0.35)	-3.57 (-3.87/-3.27)
Saint Vincent and the Grenadines	0 (0-0)	0.19 (0.02-0.45)	0 (0-1)	0.16 (0.02-0.39)	-0.43 (-0.57/-0.29)
Samoa	0 (0-1)	0.6 (0.06-1.32)	1 (0-2)	0.64 (0.06-1.35)	0.25 (0.21/0.28)
San Marino	1 (0-1)	1.52	0 (0-1)	0.56	-2.33

		(0.16-3.08)		(0.07-1.26)	(-2.64/-2.01)
Sao Tome and Principe	0 (0-0)	0.13 (0.01-0.32)	0 (0-0)	0.12 (0.01-0.27)	-0.65 (-0.85/-0.45)
Saudi Arabia	23 (3-45)	0.41 (0.05-0.81)	73 (9-150)	0.39 (0.05-0.8)	-0.22 (-0.42/-0.02)
Senegal	11 (1-24)	0.35 (0.04-0.79)	22 (2-53)	0.31 (0.03-0.72)	-0.43 (-0.61/-0.25)
Serbia	310 (41-637)	2.68 (0.36-5.53)	376 (41-842)	2.32 (0.26-5.22)	-0.3 (-0.67/0.07)
Seychelles	1 (0-1)	0.98 (0.11-2.12)	1 (0-2)	0.64 (0.07-1.47)	-1.53 (-1.71/-1.36)
Sierra Leone	3 (0-7)	0.17 (0.02-0.33)	5 (1-10)	0.14 (0.01-0.28)	-0.43 (-0.58/-0.29)
Singapore	25 (2-52)	1.15 (0.11-2.41)	31 (3-69)	0.36 (0.04-0.81)	-3.74 (-3.93/-3.55)
Slovakia	146 (16-310)	2.45 (0.27-5.19)	119 (11-262)	1.26 (0.12-2.76)	-1.97 (-2.14/-1.8)
Slovenia	63 (8-117)	2.53 (0.33-4.69)	66 (7-131)	1.5 (0.15-3)	-1.49 (-1.69/-1.28)
Solomon Islands	1 (0-3)	0.86 (0.09-2.13)	3 (0-8)	0.98 (0.11-2.27)	0.55 (0.27/0.83)
Somalia	3 (0-7)	0.11 (0.01-0.28)	6 (1-15)	0.09 (0.01-0.24)	-0.52 (-0.59/-0.45)
South Africa	198 (23-384)	0.94 (0.11-1.82)	257 (33-495)	0.54 (0.07-1.05)	-1.87 (-2.06/-1.68)
South Sudan	3 (0-8)	0.13 (0.02-0.3)	5 (1-11)	0.13 (0.01-0.28)	-0.08 (-0.18/0.02)
Spain	1053 (126-2095)	1.98 (0.24-3.94)	857 (95-1893)	0.94 (0.1-2.1)	-2.46 (-2.7/-2.21)

Sri Lanka	26 (3-56)	0.25 (0.03-0.54)	46 (5-93)	0.17 (0.02-0.34)	-1.22 (-1.34/-1.09)
Sudan	43 (5-95)	0.47 (0.05-1.05)	94 (10-199)	0.49 (0.05-1.03)	0.21 (0.16/0.26)
Suriname	2 (0-4)	0.68 (0.07-1.53)	3 (0-7)	0.39 (0.04-1.09)	-1.9 (-2.26/-1.53)
Sweden	102 (13-210)	0.75 (0.1-1.55)	77 (9-159)	0.36 (0.04-0.75)	-2.19 (-2.4/-1.98)
Switzerland	123 (12-250)	1.28 (0.13-2.59)	79 (8-177)	0.46 (0.04-1.03)	-3.28 (-3.36/-3.2)
Syrian Arab Republic	49 (5-96)	0.93 (0.09-1.87)	90 (9-198)	0.69 (0.06-1.51)	-1.26 (-1.42/-1.11)
Taiwan (Province of China)	295 (37-563)	1.89 (0.24-3.61)	656 (80-1384)	1.54 (0.19-3.23)	-0.83 (-1.15/-0.5)
Tajikistan	34 (4-63)	1.2 (0.15-2.23)	24 (3-55)	0.39 (0.05-0.91)	/-3.42 (-3.68/-3.15)
Thailand	450 (56-1048)	1.33 (0.17-3.09)	798 (96-1855)	0.74 (0.09-1.71)	-2.57 (-2.77/-2.37)
Timor-Leste	1 (0-3)	0.47 (0.06-1.09)	4 (0-9)	0.5 (0.05-1.12)	0.16 (-0.01/0.33)
Togo	3 (0-7)	0.24 (0.03-0.55)	10 (1-22)	0.24 (0.02-0.54)	0.04 (-0.03/0.11)
Tokelau	0 (0-0)	0.94 (0.1-2.25)	0 (0-0)	0.94 (0.09-2.54)	-0.03 (-0.09/0.03)
Tonga	1 (0-2)	1.36 (0.13-3.35)	1 (0-3)	1.49 (0.14-3.83)	0.22 (0.02/0.43)
Trinidad and Tobago	3 (0-7)	0.4 (0.04-0.9)	5 (0-11)	0.24 (0.02-0.56)	-2.03 (-2.24/-1.81)
Tunisia	72 (8-142)	1.45 (0.17-2.	143 (14-312)	1.05 (0.1-2.3	-1.52 (-1.72/-1.

		84)		1)	31)
Turkey	1268 (131-2448)	3.7 (0.39-7.14)	2045 (213-4246)	2.18 (0.23-4.51)	-1.91 (-2.15/-1.66)
Turkmenistan	25 (3-47)	1.22 (0.15-2.34)	25 (3-53)	0.58 (0.06-1.23)	-2.32 (-2.7/-1.93)
Tuvalu	0 (0-0)	1.03 (0.1-2.59)	0 (0-0)	1.12 (0.12-2.65)	0.15 (0.07/0.23)
Uganda	5 (1-10)	0.07 (0.01-0.15)	10 (1-22)	0.07 (0.01-0.15)	-0.89 (-1.27/-0.51)
Ukraine	1507 (182-3029)	2.06 (0.25-4.13)	535 (67-1155)	0.7 (0.09-1.52)	-3.73 (-3.91/-3.55)
United Arab Emirates	6 (1-12)	1.37 (0.14-2.84)	28 (4-57)	0.94 (0.12-1.94)	-0.03 (-0.51/0.45)
United Kingdom	1697 (206-3281)	2 (0.24-3.83)	594 (82-1217)	0.48 (0.07-0.99)	-4.62 (-4.68/-4.55)
United Republic of Tanzania	14 (2-28)	0.13 (0.01-0.26)	25 (3-52)	0.1 (0.01-0.21)	-1.26 (-1.36/-1.15)
United States of America	6175 (736-11968)	2.03 (0.24-3.92)	3984 (523-7939)	0.68 (0.09-1.36)	-3.75 (-3.91/-3.59)
United States Virgin Islands	0 (0-1)	0.56 (0.05-1.28)	1 (0-2)	0.39 (0.04-0.87)	-1.04 (-1.22/-0.86)
Uruguay	76 (8-158)	1.99 (0.21-4.12)	65 (8-134)	1.25 (0.16-2.57)	-1.89 (-2.1/-1.69)
Uzbekistan	73 (8-169)	0.61 (0.07-1.43)	83 (9-193)	0.3 (0.03-0.7)	-2.2 (-2.44/-1.96)
Vanuatu	0 (0-1)	0.42 (0.05-0.97)	1 (0-1)	0.39 (0.05-0.82)	-0.4 (-0.47/-0.32)
Venezuela (Bolivarian Republic of)	58 (6-115)	0.61 (0.07-1.22)	115 (13-235)	0.39 (0.04-0.78)	-1.51 (-1.65/-1.38)
Viet Nam	369	0.92	1036	1.03	-0.01

	(40-914)	(0.1-2.28)	(111-2368)	(0.11-2.37)	(-0.31/0.3)
Yemen	30 (4-69)	0.61 (0.07-1.42)	95 (9-219)	0.71 (0.07-1.62)	0.67 (0.54/0.8)
Zambia	4 (0-9)	0.15 (0.02-0.3)	12 (1-25)	0.17 (0.02-0.34)	0.16 (0.04/0.29)
Zimbabwe	15 (2-35)	0.36 (0.04-0.88)	27 (3-67)	0.37 (0.03-0.93)	0.1 (-0.39/0.6)

Supplemental Table 2. The number of DALYs cases and the age-standardized DALYs rate of secondhand smoke-related TBL cancer at Country levels in 1990 and 2021, and its trends from 1990 to 2021. Abbreviations: TBL cancer, tracheal, bronchus, and lung cancer; DALYs, disability-adjusted life years.

	Number of DALYs cases (95% UI) in 1990	The age-standardized DALYs rate/100000 (95% UI) in 1990	Number of DALYs cases (95% UI) in 2021	The age-standardized DALYs rate/100000 (95% UI) in 2021	EAPC (95% CI)
Country					
Afghanistan	854 (78-2280)	11.52 (1.05-30.4)	1477 (142-3554)	12.34 (1.19-29.4)	0.6 (0.45/0.75)
Albania	1077 (125-2141)	49.24 (5.83-97.94)	1519 (168-3145)	35.61 (3.92-73.67)	-0.94 (-1.21/-0.66)
Algeria	1890 (228-3715)	14.9 (1.8-29.38)	4632 (572-9417)	12.33 (1.54-24.96)	-0.47 (-0.65/-0.29)
American Samoa	8 (1-18)	32.13 (3.43-76.92)	15 (1-36)	29.55 (2.8-72.19)	-0.23 (-0.29/-0.17)

Andorra	31 (4-74)	53.21 (6.05-125.37)	33 (4-73)	21.46 (2.31-47.57)	-2.7 (-2.9/-2.5)
Angola	301 (38-715)	6.2 (0.77-14.7)	1051 (126-2326)	7.08 (0.85-15.55)	0.69 (0.34/1.04)
Antigua and Barbuda	4 (1-9)	7.79 (1.02-16.96)	5 (1-13)	4.92 (0.47-11.67)	-1.47 (-1.75/-1.19)
Argentina	15038 (1741-32031)	46.16 (5.36-98.29)	11013 (1420-25282)	20.58 (2.68-47.34)	-2.46 (-2.77/-2.16)
Armenia	2021 (232-4004)	66.52 (7.59-131.58)	1836 (251-3532)	42.21 (5.8-80.63)	-1.28 (-1.52/-1.04)
Australia	6377 (621-13629)	33.6 (3.32-71.78)	4870 (491-10619)	12.06 (1.22-25.89)	-3.24 (-3.34/-3.14)
Austria	3570 (398-7572)	34.49 (3.89-72.88)	4004 (550-7801)	25.57 (3.51-49.78)	-0.68 (-1.03/-0.32)
Azerbaijan	2367 (291-4739)	42.24 (5.23-85.34)	3203 (386-6428)	27.26 (3.4-54.54)	-1.08 (-1.35/-0.8)
Bahamas	20 (2-45)	12.27 (1.5-27.19)	42 (4-93)	9.65 (0.94-21.26)	-0.39 (-0.53/-0.25)
Bahrain	95 (12-189)	50.81 (6.39-99.89)	261 (32-511)	25.94 (3.26-51.59)	-2.7 (-2.96/-2.43)
Bangladesh	3343 (432-7451)	6.72 (0.87-14.99)	7234 (761-16578)	5.02 (0.53-11.49)	-1.16 (-1.41/-0.91)
Barbados	12 (1-27)	4.51 (0.48-10.52)	12 (1-32)	2.43 (0.22-6.29)	-2.13 (-2.4/-1.85)
Belarus	4884 (490-10226)	37.09 (3.71-76.96)	3258 (320-6900)	20.96 (2.08-44.38)	-2.39 (-2.61/-2.17)
Belgium	8892 (1158-17643)	63.84 (8.38-125.97)	4943 (581-10050)	25.07 (2.96-49.99)	-2.79 (-3.04/-2.53)
Belize	6 (1-14)	6.11 (0.64-14.	15 (2-33)	4.52 (0.5-10.	-0.88 (-1.16/-

		53)		17)	0.6)
Benin	53 (6-123)	2.57 (0.29-5.99)	96 (9-219)	1.7 (0.17-3.87)	-1.44 (-1.51/-1.36)
Bermuda	17 (1-38)	26.3 (2.34-60.5)	19 (2-45)	15.17 (1.21-34.67)	-1.63 (-1.8/-1.46)
Bhutan	15 (2-34)	5.21 (0.61-12.03)	29 (3-77)	4.56 (0.52-12.21)	-0.51 (-0.69/-0.33)
Bolivia (Plurinational State of)	244 (29-494)	7.11 (0.84-14.4)	449 (49-958)	4.68 (0.51-9.99)	-1.21 (-1.52/-0.9)
Bosnia and Herzegovina	2791 (302-5496)	59.75 (6.49-116.94)	3429 (448-7109)	56.58 (7.39-118.43)	0.07 (-0.26/0.4)
Botswana	112 (11-259)	18.12 (1.77-42.31)	184 (21-425)	11.12 (1.32-25.73)	-2.05 (-2.45/-1.65)
Brazil	25134 (3072-48037)	26.4 (3.22-50.53)	33692 (4046-67710)	13.12 (1.57-26.34)	-2.48 (-2.58/-2.38)
Brunei Darussalam	54 (7-105)	47.74 (6.32-93.39)	76 (7-166)	18.97 (1.75-41.57)	-2.63 (-2.89/-2.37)
Bulgaria	7821 (963-14723)	63.16 (7.88-118.94)	6150 (669-13228)	50.42 (5.49-108.65)	-0.43 (-0.63/-0.23)
Burkina Faso	131 (14-276)	2.85 (0.32-5.97)	258 (28-579)	2.61 (0.29-5.95)	-0.15 (-0.4/0.11)
Burundi	75 (8-161)	2.93 (0.32-6.29)	87 (10-199)	1.43 (0.16-3.27)	-2.69 (-3.09/-2.29)
Cabo Verde	13 (1-31)	5.7 (0.56-14.14)	31 (3-69)	6.61 (0.67-14.9)	0.09 (-0.27/0.44)
Cambodia	1108 (107-2505)	22.72 (2.15-51.32)	4056 (383-8497)	30.48 (2.86-64.13)	0.82 (0.67/0.97)
Cameroon	160 (17-384)	3.33 (0.36-7.96)	366 (35-884)	2.62 (0.25-6.33)	-0.78 (-0.9/-0.67)
Canada	23206	74.6	13644	20.53	-4.17

	(2487-43957)	(8.03-141.38)	(1395-27813)	(2.12-41.49)	(-4.32/-4.02)
Central African Republic	76 (8-194)	5.51 (0.54-14.06)	116 (10-333)	4.02 (0.34-11.17)	-1.21 (-1.4/-1.03)
Chad	60 (7-145)	2.06 (0.22-4.97)	168 (19-409)	2.63 (0.3-6.35)	0.82 (0.75/0.89)
Chile	2361 (259-5170)	22.87 (2.51-50.08)	2797 (344-5964)	10.88 (1.34-23.2)	-2.33 (-2.4/-2.26)
China	581552 (72614-1097292)	65.04 (8.06-122.56)	1359730 (170188-2537368)	63.32 (7.95-117.85)	-0.17 (-0.28/-0.07)
Colombia	1954 (221-4020)	10.52 (1.18-21.59)	2283 (256-4765)	4.13 (0.46-8.63)	-3.41 (-3.56/-3.26)
Comoros	11 (1-25)	5.24 (0.56-11.54)	23 (3-53)	4.4 (0.52-10.03)	-0.9 (-1.04/-0.76)
Congo	102 (13-244)	8.48 (1.06-19.94)	217 (21-531)	6.59 (0.65-15.89)	-1.22 (-1.52/-0.92)
Cook Islands	4 (1-10)	34.98 (4.62-80.7)	7 (1-17)	26.05 (2.97-65.36)	-1.13 (-1.26/-1)
Costa Rica	168 (17-413)	9.53 (0.95-23.48)	256 (25-638)	4.61 (0.45-11.53)	-2.33 (-2.5/-2.15)
C?te d’Ivoire	187 (18-431)	4.17 (0.4-9.69)	355 (39-893)	2.83 (0.31-7.19)	-2.43 (-3.13/-1.72)
Croatia	6413 (907-12028)	98.07 (13.8-183.92)	4520 (530-9454)	55.99 (6.57-117.72)	-1.36 (-1.54/-1.17)
Cuba	4678 (567-9708)	45.52 (5.51-94.24)	5772 (606-12513)	29.88 (3.15-64.61)	-1.47 (-1.7/-1.24)
Cyprus	230 (26-449)	28.62 (3.22-56.7)	443 (59-889)	22.27 (2.96-44.82)	-0.19 (-0.43/0.06)
Czechia	10366 (1159-20993)	79.04 (8.88-160.03)	6154 (710-12366)	30.97 (3.61-61.99)	-2.95 (-3.03/-2.87)

Democratic People's Republic of Korea	8901 (1018-18049)	50.04 (5.83-102.18)	13776 (1112-32201)	40.06 (3.22-93.62)	-0.74 (-0.83/-0.65)
Democratic Republic of the Congo	629 (65-1340)	3.54 (0.37-7.62)	1147 (121-2783)	2.63 (0.27-6.32)	-1.11 (-1.45/-0.77)
Denmark	5447 (613-11180)	77.17 (8.7-158.48)	2196 (272-4548)	20.75 (2.58-42.65)	-4.38 (-4.5/-4.27)
Djibouti	11 (1-25)	6.82 (0.79-16.13)	50 (5-102)	6.87 (0.74-13.99)	-0.02 (-0.05/0.02)
Dominica	5 (1-13)	9.12 (1.01-22.18)	6 (1-16)	7.3 (0.83-18.07)	-0.69 (-0.95/-0.42)
Dominican Republic	387 (36-842)	9.91 (0.91-21.77)	878 (70-2242)	8.58 (0.68-21.93)	-0.21 (-0.48/0.06)
Ecuador	263 (31-582)	4.66 (0.54-10.31)	383 (50-766)	2.3 (0.3-4.6)	-2.39 (-2.64/-2.14)
Egypt	3772 (395-7334)	11.33 (1.2-22.05)	16066 (1757-33367)	21.9 (2.41-45.84)	2.91 (2.45/3.37)
El Salvador	102 (10-238)	3.27 (0.33-7.69)	279 (27-652)	4.6 (0.45-10.76)	1.11 (0.89/1.33)
Equatorial Guinea	17 (2-41)	7.62 (0.79-18.49)	49 (6-111)	8.36 (0.95-18.8)	0.45 (0.29/0.61)
Eritrea	77 (8-208)	5.1 (0.53-13.77)	170 (16-445)	4.83 (0.45-12.71)	-0.29 (-0.45/-0.13)
Estonia	1147 (125-2396)	56.06 (6.11-117.42)	513 (58-1099)	21.54 (2.42-46.13)	-3.22 (-3.48/-2.96)
Eswatini	31 (4-69)	9.5 (1.11-21.31)	63 (7-138)	9.44 (1.13-20.76)	0.12 (-0.49/0.74)
Ethiopia	1067 (136-2252)	4.53 (0.58-9.39)	982 (125-1912)	1.97 (0.25-3.88)	-3.09 (-3.34/-2.83)
Fiji	30 (4-58)	8.09 (0.94-15.	53 (6-109)	6.66 (0.76-13	-0.74 (-1.04/-

		4)		.63)	0.44)
Finland	1438 (156-3210)	21.61 (2.34-48.05)	908 (94-2101)	8.83 (0.93-20.03)	-2.48 (-2.67/-2.28)
France	24566 (2496-56289)	34.92 (3.6-79.81)	20427 (2148-47106)	19.41 (2.04-44.32)	-1.82 (-2.13/-1.5)
Gabon	44 (5-89)	7.2 (0.79-14.66)	90 (9-205)	7.37 (0.74-16.76)	-0.03 (-0.14/0.08)
Gambia	10 (1-25)	2.65 (0.24-6.67)	22 (2-58)	2.14 (0.21-5.64)	-0.98 (-1.23/-0.73)
Georgia	3138 (382-6399)	47.91 (5.75-97.93)	2587 (309-5125)	45.41 (5.38-89.6)	1.21 (0.77/1.65)
Germany	45958 (5487-92520)	40.51 (4.9-81.43)	30896 (3216-66680)	19.72 (2.12-42.48)	-2.25 (-2.3/-2.21)
Ghana	98 (13-197)	1.38 (0.19-2.78)	261 (30-584)	1.41 (0.16-3.17)	0.29 (0.17/0.42)
Greece	9886 (1293-19079)	66.99 (8.78-129.02)	8429 (794-16968)	43.15 (4.13-86.94)	-1.44 (-1.62/-1.25)
Greenland	66 (7-142)	163.49 (16.03-355.98)	49 (5-116)	62.29 (6.26-148.42)	-3.32 (-3.49/-3.14)
Grenada	5 (1-12)	7.45 (0.84-18.13)	5 (1-11)	4.05 (0.44-9.07)	-1.84 (-2.09/-1.58)
Guam	27 (3-65)	34.45 (4.25-80.45)	55 (5-136)	25.84 (2.5-64.32)	-0.56 (-0.8/-0.32)
Guatemala	143 (18-274)	3.71 (0.46-7.08)	262 (29-510)	2.27 (0.25-4.4)	-2.11 (-2.38/-1.84)
Guinea	144 (14-366)	4.2 (0.4-10.61)	307 (25-778)	5.01 (0.41-12.72)	0.58 (0.48/0.68)
Guinea-Bissau	15 (1-39)	3.45 (0.33-8.93)	41 (3-98)	4.75 (0.36-11.58)	1.58 (1.16/2)
Guyana	24 (3-56)	5.84	26 (3-64)	3.66	-1.36

		(0.63-13.79)		(0.36-9.09)	(-1.57/-1.15)
Haiti	180 (20-394)	5.1 (0.57-11.21)	229 (24-497)	2.85 (0.3-6.26)	-1.79 (-2.07/-1.51)
Honduras	254 (26-633)	11.49 (1.16-28.59)	1102 (107-2991)	16.51 (1.61-44.63)	1.32 (1.17/1.47)
Hungary	13073 (1671-25217)	94.16 (12.03-182.23)	8685 (891-18199)	51.32 (5.2-107.55)	-2.17 (-2.52/-1.81)
Iceland	107 (13-204)	40.58 (4.8-77.38)	91 (11-184)	17.6 (2.1-35.69)	-2.7 (-2.79/-2.6)
India	28196 (3678-54865)	5.44 (0.71-10.61)	78460 (10723-154773)	6.17 (0.85-12.18)	-0.01 (-0.2/0.19)
Indonesia	17434 (1952-35795)	15.99 (1.8-33.06)	60389 (7659-121928)	23.18 (2.89-46.74)	1.2 (1.1/1.3)
Iran (Islamic Republic of)	3378 (414-6699)	11.52 (1.4-22.87)	8474 (1128-16599)	10.03 (1.34-19.71)	-0.19 (-0.39/0.02)
Iraq	2756 (321-5189)	34 (3.96-63.8)	8276 (875-16829)	33.16 (3.53-67.26)	-0.27 (-0.39/-0.16)
Ireland	2129 (244-4458)	54.28 (6.19-113.16)	1217 (130-2702)	16.33 (1.74-36.5)	-3.87 (-4.08/-3.67)
Israel	1419 (176-2897)	31.3 (3.86-63.68)	1607 (185-3676)	14.35 (1.65-32.82)	-2.35 (-2.7/-2)
Italy	49426 (5708-93090)	59.56 (6.9-112.81)	26431 (3308-51899)	21.82 (2.72-42.82)	-3.28 (-3.35/-3.21)
Jamaica	274 (28-569)	16.21 (1.65-33.64)	327 (35-736)	10.57 (1.12-23.81)	-1.95 (-2.37/-1.53)
Japan	50893 (6762-97124)	29.67 (3.95-56.61)	44789 (5761-89750)	14.08 (1.85-28.37)	-2.59 (-2.82/-2.36)
Jordan	442 (57-829)	29.23 (3.73-55.16)	1440 (164-3075)	16.87 (1.92-35.9)	-1.82 (-2.1/-1.53)

Kazakhstan	8111 (915-17783)	58.81 (6.54-128.88)	3464 (376-7507)	17.55 (1.93-38.09)	-3.82 (-4.01/-3.63)
Kenya	97 (11-208)	1.09 (0.13-2.32)	298 (35-589)	1.13 (0.13-2.25)	-0.07 (-0.33/0.18)
Kiribati	7 (1-16)	17.91 (2.1-40.36)	21 (3-50)	26.58 (3.49-63.61)	1.15 (0.8/1.5)
Kuwait	168 (20-315)	25.2 (3.11-47.73)	401 (53-770)	12.05 (1.61-23.13)	-2.04 (-2.35/-1.73)
Kyrgyzstan	1384 (159-2725)	43.58 (5.06-85.76)	1053 (133-2094)	19.6 (2.46-38.94)	-1.9 (-2.28/-1.51)
Lao People's Democratic Republic	671 (76-1700)	30.17 (3.4-76.25)	1006 (105-2462)	20.36 (2.14-49.68)	-1.46 (-1.54/-1.38)
Latvia	1885 (178-4110)	52.56 (4.99-114.11)	690 (73-1593)	19.82 (2.06-46.09)	-3.26 (-3.48/-3.04)
Lebanon	1007 (142-2006)	43.67 (6.13-87.33)	2375 (235-4678)	40.58 (4-80.22)	0.42 (0.06/0.78)
Lesotho	115 (10-282)	12.87 (1.13-31.41)	382 (31-863)	31.83 (2.62-72.21)	3.76 (3.28/4.25)
Liberia	24 (3-50)	2.01 (0.21-4.19)	39 (4-83)	1.59 (0.16-3.36)	-0.62 (-0.85/-0.38)
Libya	928 (118-1834)	47.04 (6.05-93)	2838 (347-5956)	49.21 (6-103.03)	0.38 (0.13/0.64)
Lithuania	1764 (167-3880)	38.84 (3.7-85.28)	919 (85-2045)	18.2 (1.65-40.43)	-2.66 (-2.8/-2.52)
Luxembourg	277 (25-577)	53.8 (4.86-111.78)	222 (19-479)	22.1 (1.89-47.68)	-2.8 (-2.95/-2.66)
Madagascar	237 (25-461)	4.26 (0.45-8.28)	313 (34-652)	2.26 (0.25-4.64)	-2.2 (-2.43/-1.98)
Malawi	42 (5-89)	0.97 (0.12-2.0	105 (11-249)	1.2 (0.13-2.	0.46 (0.14/0.

		4)		84)	78)
Malaysia	2106 (251-4843)	21.65 (2.58-49.95)	5610 (521-14199)	18.81 (1.76-47.32)	-0.5 (-0.83/-0.16)
Maldives	23 (2-49)	23.76 (2.5-51.2)	31 (3-72)	8.66 (0.85-19.9)	-3.95 (-4.15/-3.74)
Mali	97 (13-200)	2.23 (0.3-4.58)	300 (30-685)	3.12 (0.32-7.02)	1.32 (1.25/1.38)
Malta	147 (18-288)	34.43 (4.28-67.55)	137 (15-287)	17.45 (2-35.87)	-2.11 (-2.27/-1.96)
Marshall Islands	5 (0-11)	27.3 (2.52-66.39)	11 (1-29)	29.44 (2.59-75.73)	0.23 (0.15/0.31)
Mauritania	73 (9-179)	7.06 (0.9-17)	116 (16-254)	5.16 (0.7-11.45)	-1.17 (-1.37/-0.97)
Mauritius	119 (13-274)	15.67 (1.74-35.87)	222 (22-505)	11.82 (1.19-26.82)	-1.08 (-1.36/-0.8)
Mexico	4417 (572-8306)	10.04 (1.3-18.87)	3772 (481-7263)	2.9 (0.37-5.57)	-4.55 (-4.76/-4.33)
Micronesia (Federated States of)	15 (2-30)	28.92 (3.43-60.28)	26 (3-54)	32.12 (3.75-67.27)	0.37 (0.34/0.41)
Monaco	34 (3-74)	63.27 (6.59-137.09)	42 (4-94)	56.25 (5.72-125.19)	-0.29 (-0.59/0.01)
Mongolia	310 (40-740)	28.14 (3.7-67.42)	608 (70-1437)	23.34 (2.71-55.51)	-1.11 (-1.32/-0.9)
Montenegro	563 (73-1093)	85.63 (10.92-167.15)	828 (91-1683)	84.34 (9.36-170.96)	0.09 (-0.25/0.43)
Morocco	2966 (342-5791)	20.3 (2.34-39.58)	6624 (790-14148)	18.33 (2.17-39.29)	-0.45 (-0.64/-0.26)
Mozambique	124 (15-245)	1.96 (0.24-3.86)	277 (31-575)	2.27 (0.25-4.71)	1.01 (0.84/1.19)
Myanmar	6254	25.08	6401	12.45	-2.3

	(690-14410)	(2.76-58)	(758-12680)	(1.47-24.59)	(-2.44/-2.17)
Namibia	30 (4-59)	4.14 (0.54-8.24)	57 (7-118)	3.66 (0.46-7.48)	-0.7 (-1.03/-0.37)
Nauru	2 (0-5)	49.91 (6.25-109.36)	3 (0-6)	44.75 (5.09-89.98)	-0.48 (-0.59/-0.37)
Nepal	459 (48-959)	4.46 (0.47-9.39)	908 (116-1886)	3.71 (0.47-7.67)	-0.62 (-0.85/-0.38)
Netherlands	8527 (916-18265)	46.26 (4.95-98.69)	5911 (687-12988)	19.14 (2.21-41.98)	-2.33 (-2.56/-2.09)
New Zealand	1200 (140-2744)	32.25 (3.8-73.89)	944 (109-2220)	12.36 (1.44-29.09)	-2.99 (-3.13/-2.85)
Nicaragua	74 (8-162)	4.53 (0.49-9.89)	193 (19-461)	3.78 (0.37-8.97)	-0.34 (-0.49/-0.2)
Niger	56 (6-124)	1.83 (0.19-4.02)	125 (14-272)	1.38 (0.15-3.01)	-0.76 (-0.98/-0.54)
Nigeria	194 (22-385)	0.41 (0.05-0.81)	493 (54-994)	0.48 (0.05-0.96)	0.7 (0.56/0.84)
Niue	1 (0-1)	25.04 (2.6-60.44)	1 (0-2)	28.64 (3.26-73.3)	0.32 (0.23/0.4)
North Macedonia	1438 (180-2882)	71.95 (9.03-143.31)	2184 (241-4578)	63.26 (7.03-132.65)	-0.43 (-0.8/-0.07)
Northern Mariana Islands	8 (1-19)	44.22 (5.13-98.68)	19 (2-45)	34.17 (3.93-80.84)	-0.97 (-1.08/-0.86)
Norway	1467 (192-2795)	25.88 (3.45-49.06)	1145 (148-2303)	12.66 (1.64-25.27)	-2.48 (-2.8/-2.16)
Oman	51 (6-120)	6.97 (0.81-16.5)	103 (10-244)	4.58 (0.47-10.88)	-1.13 (-1.52/-0.73)
Pakistan	10785 (1254-22133)	18.31 (2.12-37.51)	21621 (2358-46423)	15.88 (1.73-34.37)	-0.73 (-1.12/-0.35)

Palau	4 (0-10)	40.98 (4.6-104.15)	9 (1-23)	38.84 (3.75-96.93)	-0.23 (-0.3/-0.16)
Palestine	304 (38-596)	33.81 (4.27-65.7)	768 (88-1604)	27.02 (3.06-56.01)	-0.9 (-1.08/-0.72)
Panama	153 (14-353)	10.03 (0.92-23.07)	161 (16-399)	3.65 (0.37-9.03)	-3.52 (-3.7/-3.33)
Papua New Guinea	276 (28-619)	14.49 (1.47-32.27)	953 (117-2200)	17.89 (2.18-41.6)	0.6 (0.53/0.66)
Paraguay	244 (25-575)	10.63 (1.09-25.09)	676 (69-1739)	11.29 (1.15-28.89)	0.2 (0.06/0.35)
Peru	815 (88-1506)	6.4 (0.69-11.85)	894 (97-1811)	2.59 (0.28-5.25)	-3.93 (-4.39/-3.48)
Philippines	8538 (1113-15994)	27.13 (3.55-50.54)	15890 (1924-30412)	17.94 (2.17-34.26)	-1.46 (-1.62/-1.3)
Poland	37664 (4970-71729)	86.07 (11.25-164.28)	25650 (3760-53477)	37.46 (5.53-77.4)	-2.91 (-3.14/-2.69)
Portugal	3616 (326-7476)	27.58 (2.49-56.49)	3169 (261-6998)	16.18 (1.34-36.02)	-1.81 (-2.1/-1.52)
Puerto Rico	297 (32-674)	8.27 (0.89-18.72)	251 (23-565)	4.1 (0.37-9.21)	-2.29 (-2.41/-2.16)
Qatar	47 (6-97)	37.95 (5.11-76.65)	208 (26-418)	18.33 (2.22-37.15)	-2.35 (-2.88/-1.82)
Republic of Korea	14331 (1701-28579)	43.01 (5.16-85.41)	17099 (1893-39430)	18.1 (1.99-41.72)	-3.33 (-3.66/-3)
Republic of Moldova	1932 (235-3787)	41.14 (5-80.14)	1249 (148-2496)	21.63 (2.57-43.05)	-1.63 (-1.88/-1.37)
Romania	12677 (1390-26136)	44 (4.83-90.35)	11884 (921-26998)	36.87 (2.9-84.46)	-0.81 (-1.04/-0.58)
Russian Federation	71807 (8469-1373	38.21 (4.52-72.	54191 (6289-1079	23.12 (2.65-46	-1.69 (-1.94/-

	67)	88)	80)	.14)	1.44)
Rwanda	151 (17-409)	4.65 (0.53-12.53)	254 (26-715)	3.49 (0.37-9.76)	-1.68 (-2.08/-1.28)
Saint Kitts and Nevis	3 (0-6)	7.7 (0.98-16.8)	3 (0-8)	4.01 (0.47-9.89)	-2.02 (-2.16/-1.88)
Saint Lucia	8 (1-18)	9.13 (1.01-21.21)	9 (1-22)	3.61 (0.37-8.89)	-3.45 (-3.71/-3.2)
Saint Vincent and the Grenadines	3 (0-8)	4.84 (0.52-11.18)	6 (1-14)	4.12 (0.39-9.74)	-0.38 (-0.52/-0.25)
Samoa	13 (1-29)	14.53 (1.61-32.63)	24 (2-50)	15.66 (1.55-33.05)	0.29 (0.24/0.34)
San Marino	13 (1-26)	40.74 (4.4-82.29)	9 (1-21)	15.13 (1.77-34.59)	-2.31 (-2.62/-2)
Sao Tome and Principe	2 (0-6)	3.48 (0.38-8.81)	4 (0-9)	3.11 (0.33-7.15)	-0.77 (-0.98/-0.55)
Saudi Arabia	642 (82-1292)	10.11 (1.27-20.39)	2309 (299-4678)	9.55 (1.22-19.76)	-0.23 (-0.42/-0.03)
Senegal	285 (34-647)	8.5 (1-19.08)	581 (56-1378)	7.19 (0.69-17.02)	-0.53 (-0.71/-0.34)
Serbia	8917 (1159-18275)	72.93 (9.61-149.76)	9407 (1030-21049)	61.52 (6.84-137.02)	-0.45 (-0.85/-0.04)
Seychelles	14 (2-29)	24.12 (2.76-52.21)	18 (2-41)	15.02 (1.58-33.68)	-1.71 (-1.89/-1.53)
Sierra Leone	85 (10-169)	4.07 (0.49-8.05)	128 (15-264)	3.22 (0.36-6.65)	-0.47 (-0.61/-0.32)
Singapore	683 (68-1434)	28.78 (2.85-60.61)	709 (80-1562)	8.13 (0.92-18.08)	-4.07 (-4.25/-3.89)
Slovakia	4170 (464-8838)	70.97 (7.9-150.03)	3063 (294-6671)	33.58 (3.24-73.12)	-2.25 (-2.42/-2.08)
Slovenia	1824	73.58	1497	37.15	-1.98

	(242-3383)	(9.77-135.96)	(155-2978)	(3.85-73.51)	(-2.22/-1.75)
Solomon Islands	34 (4-84)	22.09 (2.43-54.33)	103 (11-240)	25.83 (2.83-59.28)	0.66 (0.37/0.95)
Somalia	87 (11-218)	3.02 (0.37-7.63)	181 (20-486)	2.44 (0.28-6.49)	-0.59 (-0.66/-0.52)
South Africa	6007 (711-11794)	26.52 (3.13-51.81)	7427 (953-14258)	14.71 (1.88-28.25)	-1.99 (-2.18/-1.81)
South Sudan	96 (11-219)	3.54 (0.41-8.07)	150 (16-322)	3.34 (0.37-7.19)	-0.19 (-0.33/-0.04)
Spain	27779 (3300-55085)	54.62 (6.55-108.34)	20665 (2307-46253)	24.79 (2.78-55.79)	-2.64 (-2.92/-2.37)
Sri Lanka	727 (77-1558)	6.25 (0.66-13.49)	1185 (135-2389)	4.25 (0.48-8.55)	-1.18 (-1.32/-1.04)
Sudan	1205 (136-2644)	12.09 (1.36-26.41)	2716 (276-5823)	12.3 (1.27-26.1)	0.13 (0.07/0.18)
Suriname	46 (5-106)	17.2 (1.91-39.14)	67 (6-188)	10.12 (0.98-28.3)	-1.9 (-2.25/-1.55)
Sweden	2590 (330-5340)	20.83 (2.67-43)	1608 (191-3303)	8.56 (1.03-17.59)	-2.66 (-2.91/-2.42)
Switzerland	3342 (328-6719)	36.52 (3.56-73.18)	1889 (184-4219)	12.1 (1.18-26.92)	-3.6 (-3.69/-3.51)
Syrian Arab Republic	1442 (146-2859)	24.6 (2.48-48.56)	2529 (244-5546)	17.29 (1.65-38.03)	-1.42 (-1.57/-1.26)
Taiwan (Province of China)	7957 (988-15225)	46.88 (5.84-89.5)	14470 (1762-30510)	34.87 (4.24-73.07)	-1.01 (-1.27/-0.75)
Tajikistan	1008 (127-1890)	33.9 (4.3-63.4)	756 (95-1732)	10.77 (1.33-24.59)	-3.6 (-3.84/-3.35)
Thailand	12371 (1527-29007)	32.47 (4.04-75.88)	18709 (2257-43241)	17.5 (2.12-40.18)	-2.7 (-2.92/-2.48)

Timor-Leste	34 (4-78)	11.2 (1.49-26.32)	103 (11-234)	11.7 (1.22-26.58)	0.06 (-0.13/0.26)
Togo	92 (10-206)	6.57 (0.72-14.77)	306 (30-687)	6.59 (0.66-14.93)	0.16 (0.08/0.24)
Tokelau	0 (0-1)	21.68 (2.15-52.1)	0 (0-1)	21.85 (1.92-59.17)	-0.05 (-0.1/0)
Tonga	18 (2-44)	31.35 (3.05-77.9)	28 (3-71)	34.16 (3.19-88.37)	0.22 (0.03/0.41)
Trinidad and Tobago	86 (9-192)	10.06 (1.07-22.47)	124 (12-286)	6.31 (0.61-14.56)	-1.96 (-2.17/-1.74)
Tunisia	1881 (226-3681)	35.43 (4.2-69.43)	3765 (363-8070)	26.81 (2.59-57.4)	-1.34 (-1.54/-1.15)
Turkey	35250 (3617-67982)	94.58 (9.81-182.63)	51114 (5475-106541)	52.53 (5.62-108.99)	-2.15 (-2.38/-1.92)
Turkmenistan	751 (93-1430)	35.16 (4.37-67.3)	759 (82-1622)	16.42 (1.78-35.16)	-2.41 (-2.8/-2.02)
Tuvalu	2 (0-5)	25.38 (2.45-63.81)	3 (0-7)	26.8 (2.92-63.4)	0.08 (0.01/0.15)
Uganda	132 (15-267)	1.91 (0.22-3.89)	304 (39-642)	1.83 (0.23-3.83)	-1.02 (-1.44/-0.6)
Ukraine	42233 (5035-84842)	58.41 (6.96-116.95)	14616 (1876-31588)	20.09 (2.57-43.29)	-3.75 (-3.93/-3.58)
United Arab Emirates	183 (20-376)	33.91 (3.53-70.79)	903 (118-1867)	19.63 (2.43-40.7)	-0.89 (-1.33/-0.44)
United Kingdom	43602 (5285-83348)	54.77 (6.65-104.03)	14246 (1953-29417)	12.64 (1.73-25.89)	-4.79 (-4.86/-4.73)
United Republic of Tanzania	399 (45-792)	3.37 (0.39-6.7)	721 (92-1513)	2.51 (0.32-5.3)	-1.26 (-1.36/-1.15)
United States of America	164857 (19533-316	56.92 (6.7-109.	93718 (12138-184	16.97 (2.19-33	-4.11 (-4.26/-

	633)	1)	055)	.37)	3.96)
United States Virgin Islands	13 (1-30)	14.14 (1.37-32.6)	16 (1-36)	9.72 (0.89-22.31)	-0.98 (-1.14/-0.83)
Uruguay	2032 (217-4195)	54.91 (5.89-112.85)	1625 (209-3348)	33.44 (4.32-68.79)	-1.97 (-2.16/-1.79)
Uzbekistan	2261 (240-5205)	18.19 (1.94-41.87)	2527 (267-5839)	8.28 (0.87-19.09)	-2.49 (-2.74/-2.24)
Vanuatu	7 (1-16)	10.32 (1.3-24.17)	18 (2-38)	9.56 (1.1-20.22)	-0.39 (-0.47/-0.31)
Venezuela (Bolivarian Republic of)	1601 (176-3171)	15.57 (1.7-30.83)	2921 (320-6017)	9.42 (1.04-19.33)	-1.71 (-1.85/-1.57)
Viet Nam	9905 (1077-24563)	23.75 (2.58-58.68)	28418 (3000-64523)	26.35 (2.81-60.31)	0.03 (-0.24/0.3)
Yemen	880 (107-2095)	16.12 (1.94-37.76)	2681 (268-6155)	17.33 (1.7-39.79)	0.42 (0.3/0.54)
Zambia	128 (14-257)	4.03 (0.44-8.05)	385 (42-777)	4.6 (0.49-9.22)	0.23 (0.1/0.36)
Zimbabwe	417 (48-1016)	9.41 (1.09-22.78)	836 (81-2111)	10.14 (0.96-25.34)	0.34 (-0.19/0.88)

Supplemental Figure 1. Numbers and age-standardized rates of secondhand smoke-related TBL cancer-related deaths and DALYs for both sexes in 2021. (A) Numbers of deaths and DALYs of secondhand smoke-related TBL cancer for both sexes in 2021; (B) Age-standardized rates of deaths and DALYs of secondhand smoke-related TBL cancer for both sexes in 2021; Abbreviations: TBL cancer, tracheal, bronchus, and lung cancer; DALYs, disability-adjusted life years.

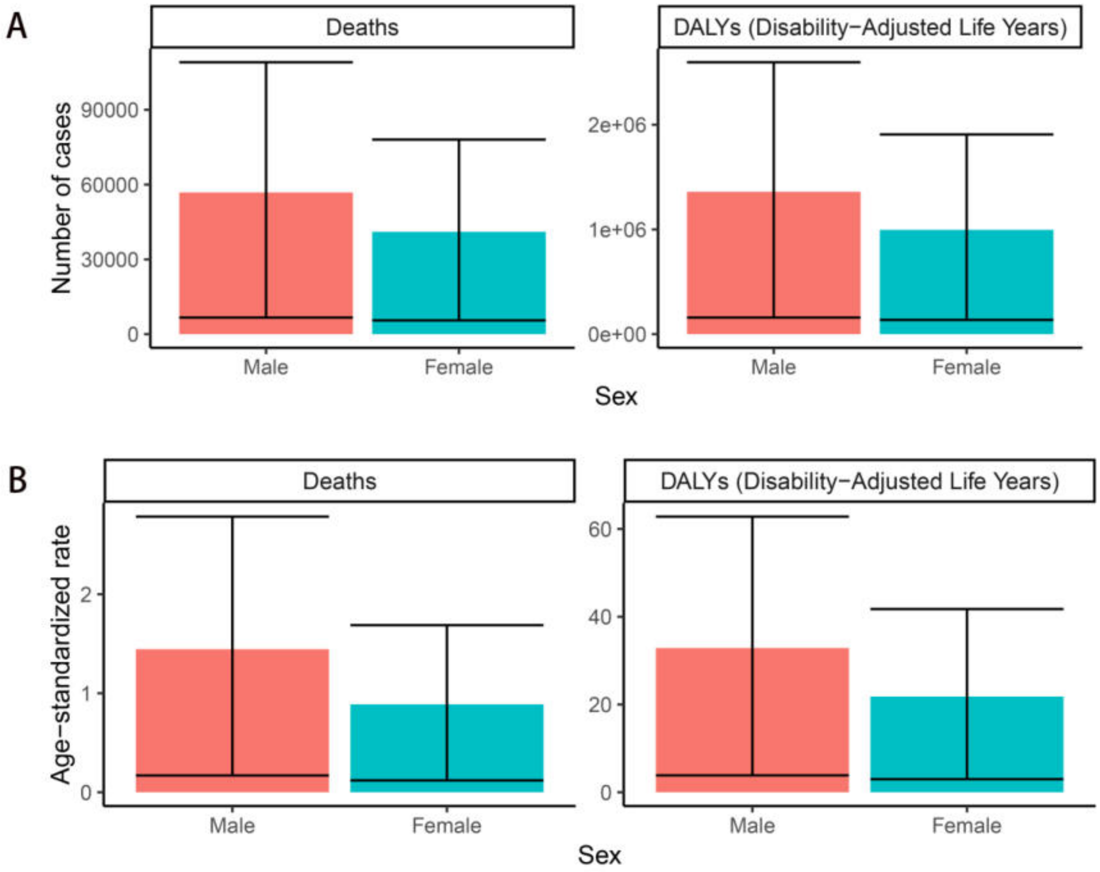

Supplemental Figure 2. Numbers and age-standardized rates of secondhand smoke-related TBL cancer-related deaths and DALYs for different age groups in 2021. (A) Numbers of deaths and DALYs of secondhand smoke-related TBL cancer for different age groups in 2021; (B) Age-standardized rates of deaths and DALYs of secondhand smoke-related TBL cancer for different age groups in 2021; Abbreviations: TBL cancer, tracheal, bronchus, and lung cancer; DALYs, disability-adjusted life years.

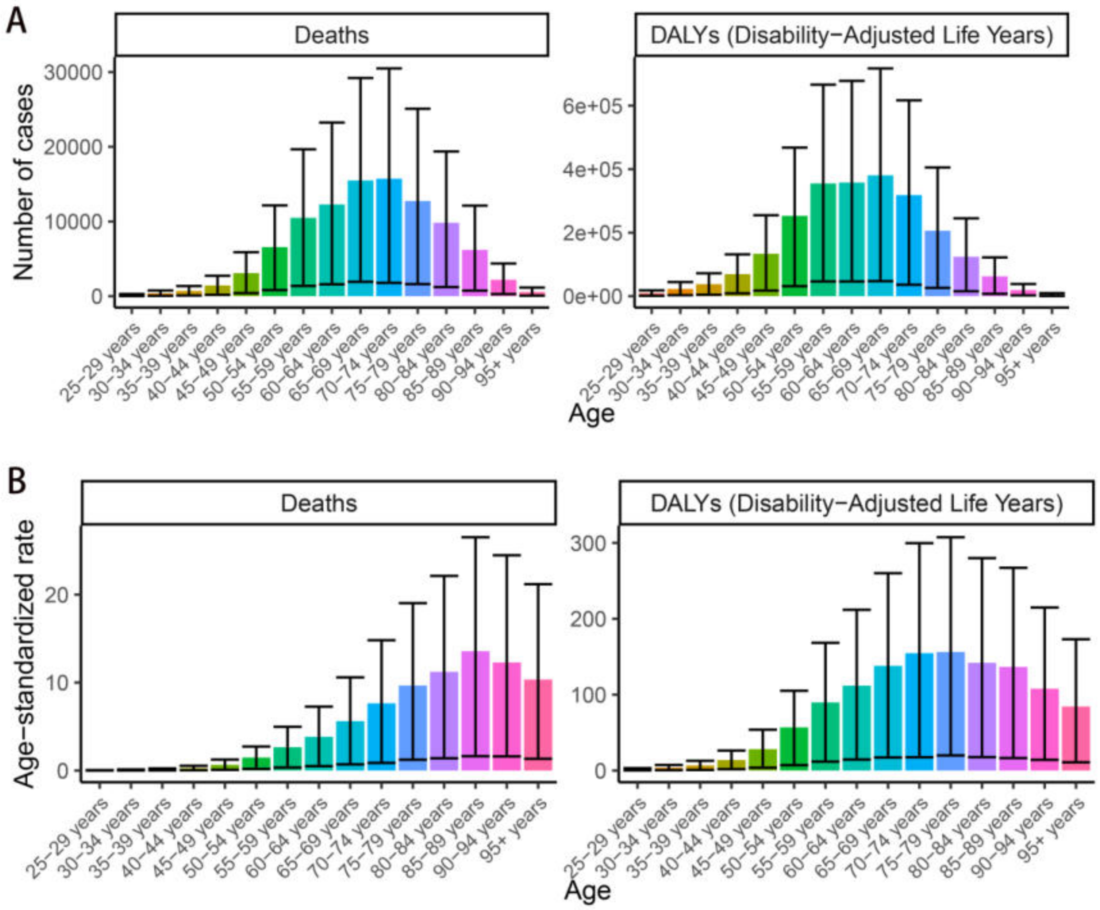

Supplemental Figure 3. Numbers and age-standardized rates of secondhand smoke-related TBL cancer-related deaths and DALYs for different SDI regions in 2021. (A) Numbers of deaths and DALYs of secondhand smoke-related TBL cancer for different SDI regions in 2021; (B) Age-standardized rates of deaths and DALYs of secondhand smoke-related TBL cancer for different SDI regions in 2021; Abbreviations: TBL cancer, tracheal, bronchus, and lung cancer; DALYs, disability-adjusted-life-years; SDI, socio-demographic index.

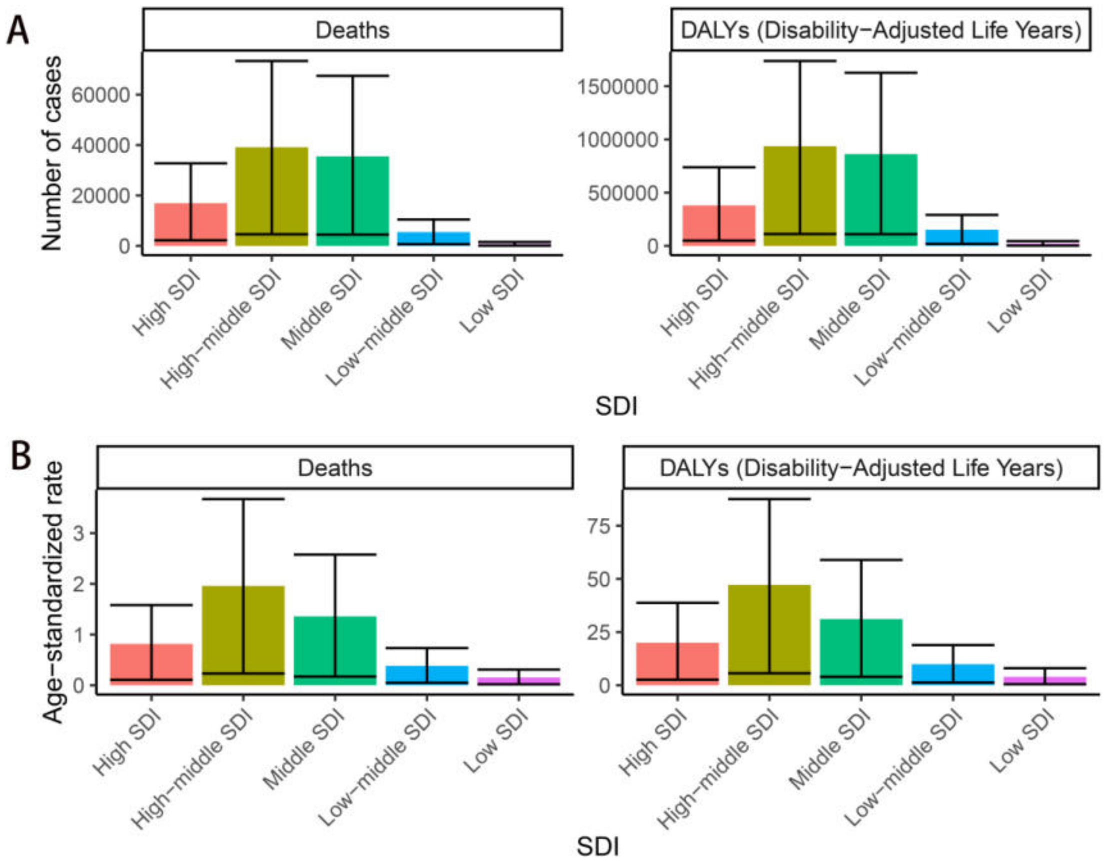

Supplemental Figure 4. Numbers and age-standardized rates of secondhand smoke-related TBL cancer-related deaths and DALYs for different GBD regions in 2021. (A) Numbers of deaths and DALYs of secondhand smoke-related TBL cancer for different GBD regions in 2021; (B) Age-standardized rates of deaths and DALYs of secondhand smoke-related TBL cancer for different GBD regions in 2021; Abbreviations: TBL cancer, tracheal, bronchus, and lung cancer; DALYs, disability-adjusted life years; GBD, Global Burden of Disease.

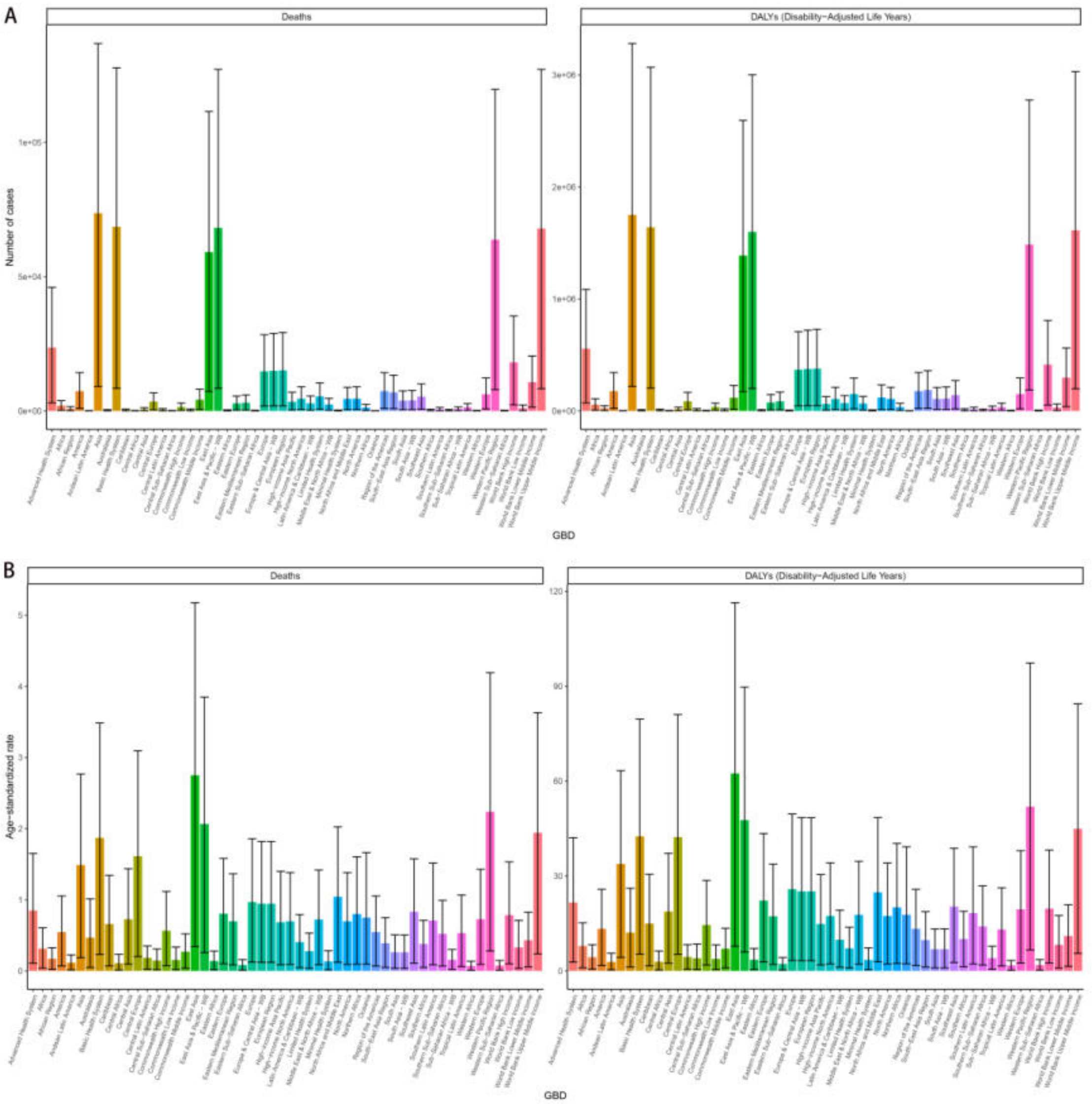

Supplemental Figure 5. Trends in the numbers and age-standardized rates of secondhand smoke-related TBL cancer-related deaths and DALYs globally by sexes from 1990 to 2021. (A) Trends in the numbers of deaths and DALYs of secondhand smoke-related TBL cancer globally by sexes from 1990 to 2021; (B) Trends in the age-standardized rates of deaths and DALYs of secondhand smoke-related TBL cancer globally by sexes from 1990 to 2021; Abbreviations: TBL cancer, tracheal, bronchus, and lung cancer; DALYs, disability-adjusted-life-years.

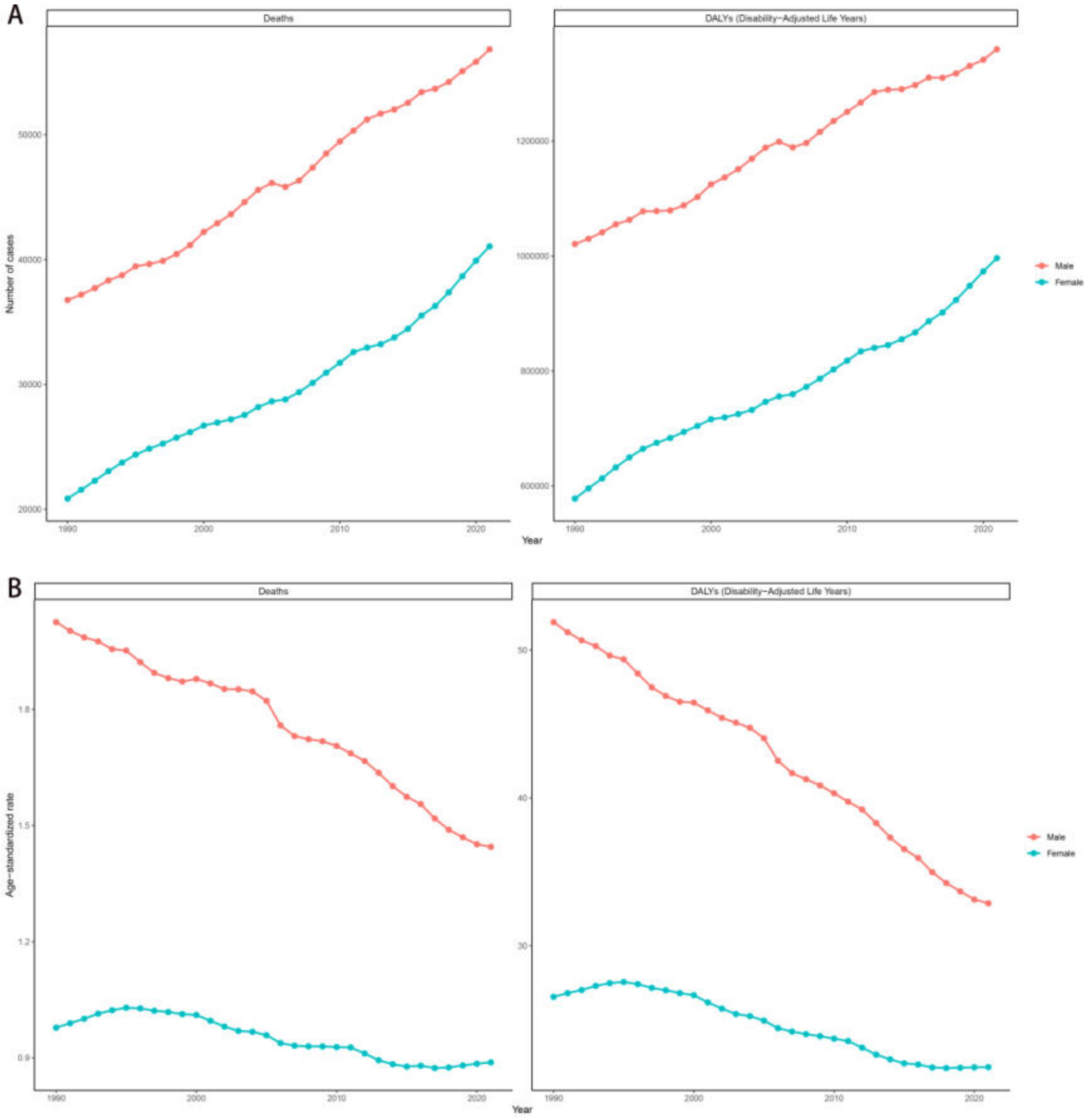

Supplemental Figure 6. Trends in the numbers and age-standardized rates of secondhand smoke-related TBL cancer-related deaths and DALYs globally by age groups from 1990 to 2021. (A) Trends in the numbers of deaths and DALYs of secondhand smoke-related TBL cancer globally by age groups from 1990 to 2021; (B) Trends in the age-standardized rates of deaths and DALYs of secondhand smoke-related TBL cancer globally by age groups from 1990 to 2021; Abbreviations: TBL cancer, tracheal, bronchus, and lung cancer; DALYs, disability-adjusted-life-years.

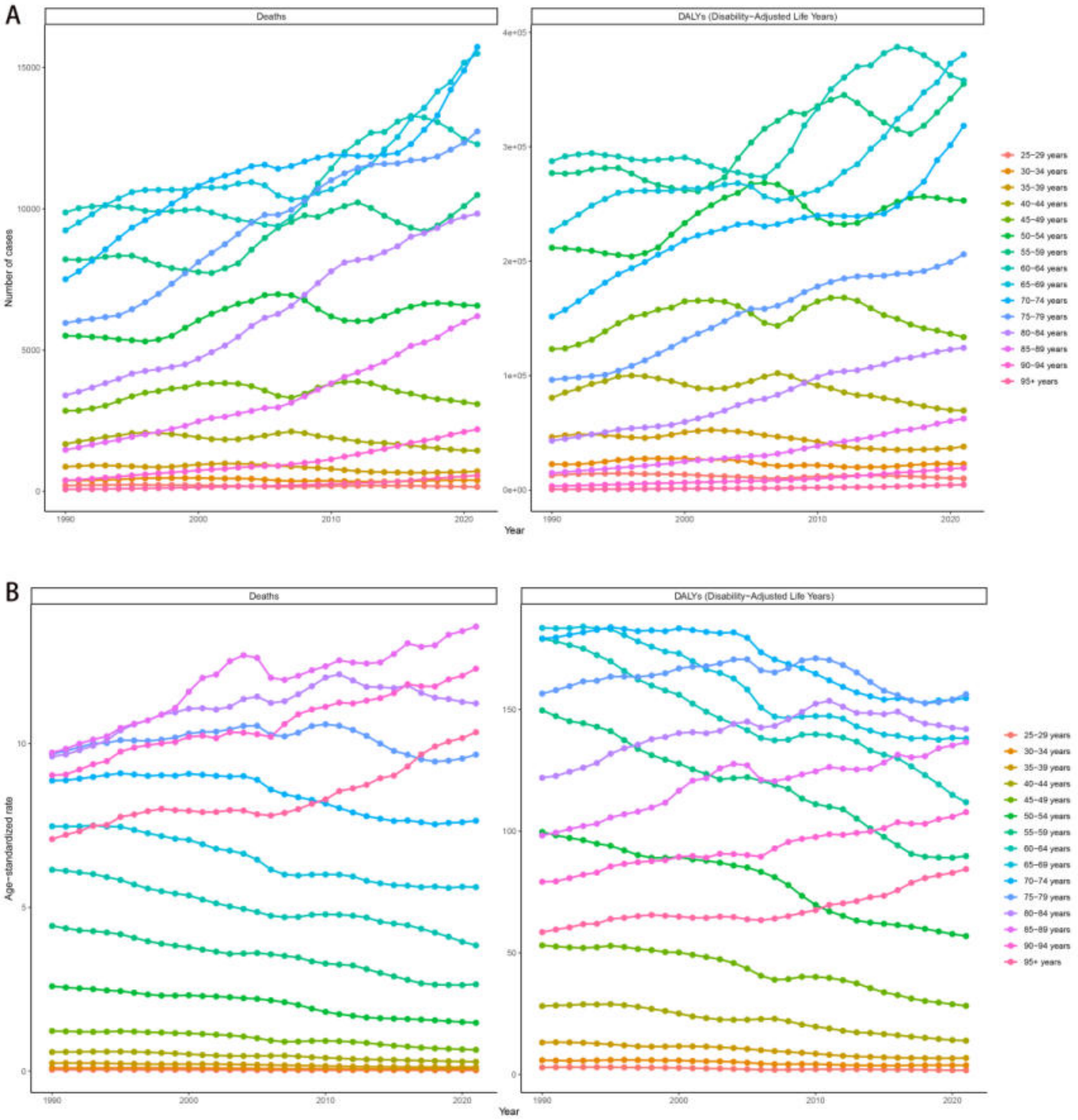

Supplemental Figure 7. Trends in the numbers and age-standardized rates of secondhand smoke-related TBL cancer-related deaths and DALYs globally by SDI regions from 1990 to 2021. (A) Trends in the numbers of deaths and DALYs of secondhand smoke-related TBL cancer globally by SDI regions from 1990 to 2021; (B) Trends in the age-standardized rates of deaths and DALYs of secondhand smoke-related TBL cancer globally by SDI regions from 1990 to 2021; Abbreviations: TBL cancer, tracheal, bronchus, and lung cancer; DALYs, disability-adjusted-life-years; SDI, socio-demographic index.

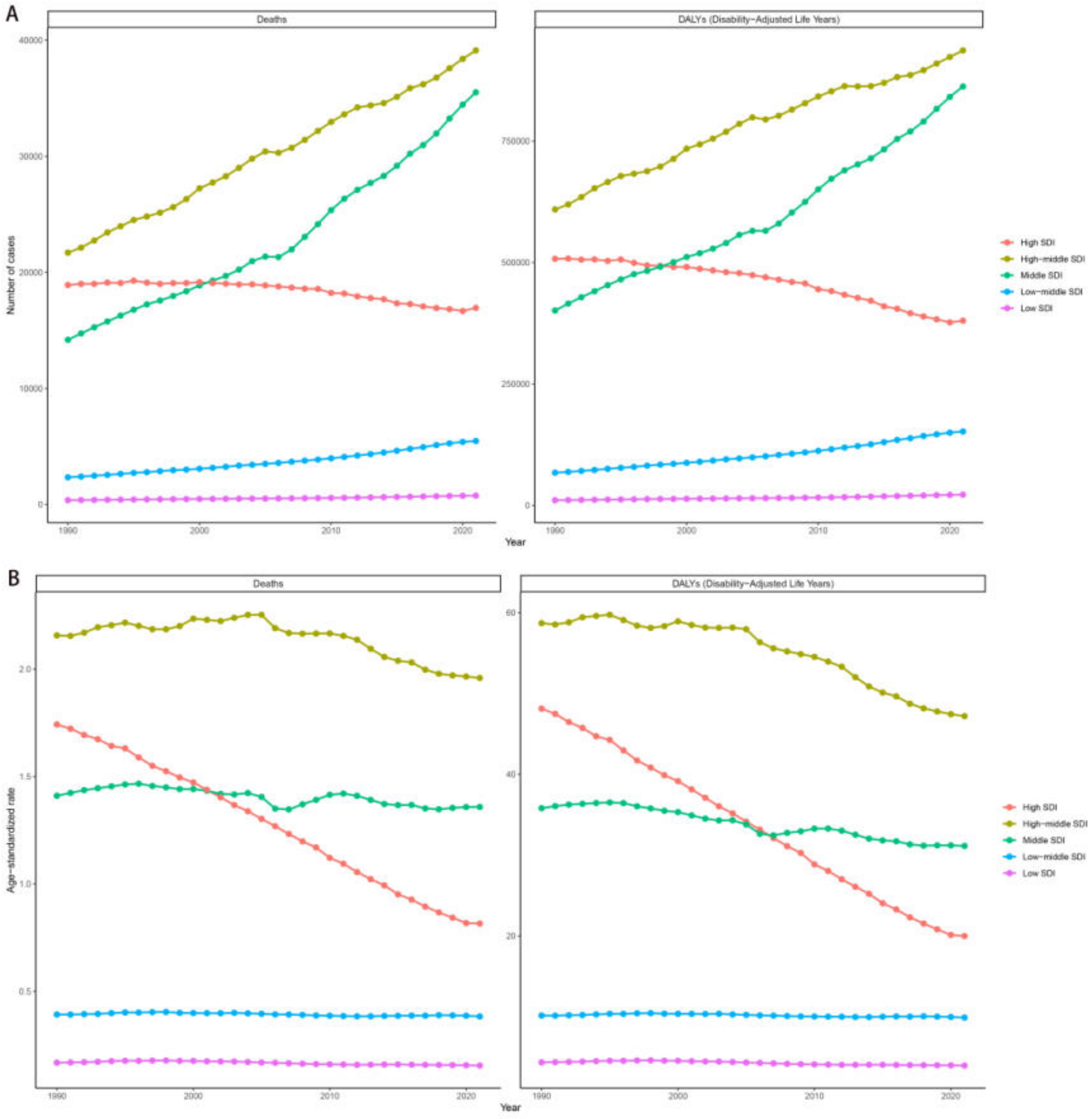

© 2025 Yao J. et al.
